# Modeling drug-induced liver injury and screening for anti-hepatofibrotic compounds using human PSC-derived organoids

**DOI:** 10.1186/s13619-022-00148-1

**Published:** 2023-03-03

**Authors:** Xiaoshan Wu, Dacheng Jiang, Yi Yang, Shuang Li, Qiurong Ding

**Affiliations:** 1 School of Biotechnology, East China University of Science and Technology, Shanghai, 200237 P. R. China; 2 CAS Key Laboratory of Nutrition, Metabolism and Food Safety, Shanghai Institute of Nutrition and Health, Shanghai Institutes for Biological Sciences, University of Chinese Academy of Sciences, Chinese Academy of Sciences, Shanghai, 200031 P. R. China; 3 School of Pharmacy, Fujian Health College, Fujian, 350101 P. R. China; 4 Shanghai Jiao Tong University Affiliated Sixth People’s Hospital, Shanghai, 200233 China; 5 Institute for Stem Cell and Regeneration, Chinese Academy of Sciences, Beijing, 100101 P. R. China

**Keywords:** Human liver organoid, Pluripotent stem cell, Drug-induced liver injury, Anti-fibrotic compounds, High-content analysis

## Abstract

Preclinical models that can accurately predict the toxicity and efficacy of candidate drugs to human liver tissue are in urgent need. Human liver organoid (HLO) derived from human pluripotent stem cells offers a possible solution. Herein, we generated HLOs, and demonstrated the utility of these HLOs in modeling a diversity of phenotypes associated with drug-induced liver injury (DILI), including steatosis, fibrosis, and immune responses. Phenotypic changes in HLOs after treatment with tool compounds such as acetaminophen, fialuridine, methotrexate, or TAK-875 showed high concordance with human clinical data in drug safety testings. Moreover, HLOs were able to model liver fibrogenesis induced by TGFβ or LPS treatment. We further devised a high-content analysis system, and established a high-throughput anti-fibrosis drug screening system using HLOs. SD208 and Imatinib were identified that can significantly suppress fibrogenesis induced by TGFβ, LPS, or methotrexate. Taken together, our studies demonstrated the potential applications of HLOs in drug safety testing and anti-fibrotic drug screening.

**Supplementary Information:**

The online version contains supplementary material available at 10.1186/s13619-022-00148-1.

## Background

The high failure rate of the new drug development has been well recognized. The majority of failures were due to a lack of either efficacy, or safety, or combined, representing in total 73% of all phase II failures and 69% of all phase III failures (Harrison 2016). The primary reason of the high failure rate is the heavy relying on the pre-clinical data obtained from animal experiments. Many studies have demonstrated biological processes that are specific to the human body and cannot be modeled with animal models. For example, the liver cytochrome P450 (CYP) enzymes are different between human beings and animals (Hammer et al. 2021), thus animals have different susceptibilities to toxic agents than human beings. An analysis of 150 drugs that caused adverse events in humans showed that the regulatory testings in rats and dogs can only correctly predict 71% of toxicities in humans (Jang et al. 2019). A recent survey comparing target organ toxicities in animal and first-in-human studies also identified a low concordance of drug-induced liver injury (DILI) between human and animals (Monticello et al. 2017). Also in other cases, many human liver pathologies occur under a specific metabolic or immune context, such as non-alcoholic steatohepatitis (NASH) and autoimmune hepatitis (AIH) (Ahmed et al. 2021; Mahdinloo et al. 2020), which cannot be fully recapitulated in animal models. Therefore, the lack of physiological relevance between human and animal models calls for an urgent need to develop human-originated in vitro cell models for studying drug toxicity and efficacy.

Human liver organoids (HLOs) generated from pluripotent stem cells (PSCs) were recently reported that contain multiple cell types, including hepatocytes, liver stellate cells, and kupffer cells (Ouchi et al. 2019). These HLOs were able to model steatohepatitis induced by free fatty acids treatment or genetic mutation of certain genes, or drug-induced cholestasis (Shinozawa et al. 2021). However, as a potential cellular model for studying drug toxicities, whether HLO can recapitulate DILI by toxins with different mechanisms has not been examined. Herein, we treated HLO with tool compounds that are known to cause diverse phenotypes of DILI, and characterized the utility of HLOs for risk assessment in humans. We found good concordance between DILI in human clinic data and in vitro models of HLOs. We further devised a high-content analysis (HCA) system for fibrogenesis analysis, and developed a high-throughput anti-fibrosis drug screening system using HLOs. Taken together, our study demonstrated that HLOs can be applied to model DILI and drug screenings.

## Results

### Functional characterization of human liver organoids derived from hPSCs

**Fig. 1:**
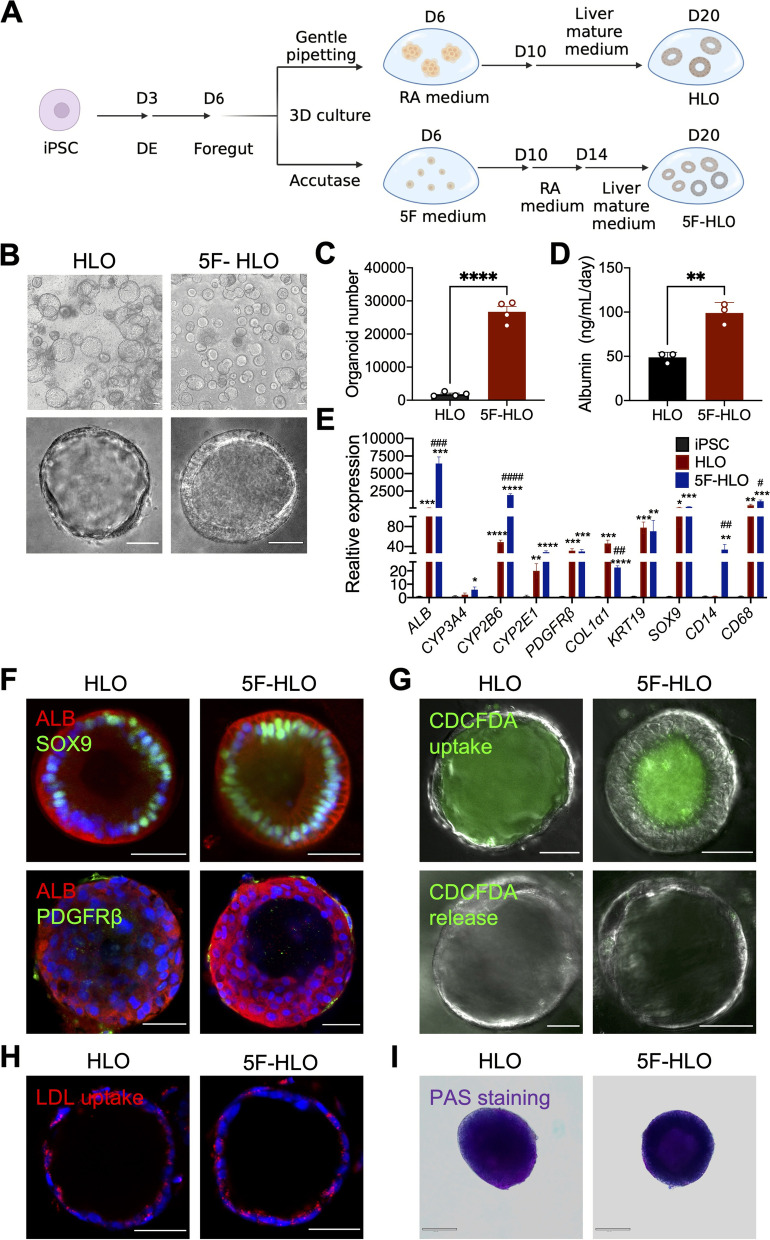
Functional characterization of human liver organoids derived from hPSCs. **A** Schematic representation of the differentiation method for human liver organoids. DE represents definitive endoderm. **B** Representative bright field image (4 × , 20 ×) of HLOs or 5F-HLOs at day 20. Scale bar, 100 μm, 50 μm, respectively. **C**, **D** The number and the albumin secretion level of HLOs or 5F-HLOs. **E** Expression level of representative genes in iPSC, HLOs and 5F-HLOs. Hepatic markers (ALB, CYP3A4, CYP2B6, CYP2E1), stellate cell markers (PDGFRβ, COL1α1), cholangiocyte markers (KRT19, SOX9), and kupffer cell markers (CD14, CD68). mRNA levels were assessed by qPCR and expressed as fold versus iPSC. *n* = 3 independent differentiations, *P < 0.05, *#P* < 0.05 versus HLO. **F** Immunostaining for Albumin (ALB), SOX9 and PDGFRβ in HLO and 5F-HLO. Nuclei were stained with DAPI (blue). Scale bar, 50 μm. **G** CDCFDA uptake and release in HLO and 5F-HLO. Scale bar, 50 μm. **H** LDL uptake evaluated by DiI-LDL fluorescent staining in HLO and 5F-HLO (DiI-LDL in red, and DAPI in blue). Scale bar, 50 μm. **I** Glycogen accumulation evaluated by PAS staining in HLO and 5F-HLO. Scale bar, 50 μm. Values represent means with SEM. *P* values were assessed by unpaired, two-tailed Student’s t test (**C**, **D**), and one-way ANOVA with Dunnett’s multiple comparisons test (**E**)

To develop HLOs from hPSCs, we followed a previously described protocol (Ouchi et al. 2019; Shinozawa et al. 2021). Human PSCs were initially differentiated to foregut spheroids, which were then cultured in 3D with retinoid acid (RA) to allow the specification of both parenchymal and non-parenchymal cells, followed by further maturation in liver maturation medium (Fig. 1A). Alternatively, foregut cells can be dissociated into single cells using accutase, and then expanded in medium that is composed of 5 factors, including fibroblast growth factor 2 (FGF2), vascular endothelial growth factor (VEGF), epidermal growth factor (EGF), CHIR99021 (glycogen synthase kinase 3 inhibitor) and A83-01 (transforming growth factor-β inhibitor) with ascorbic acid, before RA treatment and further maturation (Fig. 1A). Consistent with previous report (Shinozawa et al. 2021), this protocol with additional expansion in medium with 5 factors (therefore termed as “5F”) resulted in almost a tenfold number of HLOs than the one without 5F treatment (termed as “HLO”) (Figs. 1B, C). In addition, analyses of albumin secretion (Fig. 1D) and expression levels of several hepatic marker genes (*ALB*, *CYP3A4*, *CYP2B6*, *CYP2E1*) (Fig. 1E) suggested that 5F method-derived HLOs contain more mature hepatocytes. Gene expression analysis also demonstrated the expression of markers from stellate cells (*PDGFRβ*, *COL1α1*), cholangiocytes (*KRT19*, *SOX9*), and kupffer cells (*CD14*, *CD68*) (Fig. 1E), which were then further confirmed by immunofluorescence analysis of the cholangiocyte marker (SOX9) and stellate cell marker (PDGFRβ) (Fig. 1F), proving that multiple cell types exist in HLOs. Additional functional assessment with CDCFDA uptake and release, indicating of bile canaliculi and multi-drug resistance protein 2 (MRP2) activity (Torok et al. 2020) (Fig. 1G), DiI-LDL uptake, suggesting low-density lipoprotein uptake (Fig. 1H), and periodic acid-Schiff (PAS) staining for examination of glycogen accumulation (Fig. 1I), altogether demonstrated basic hepatocyte and cholangiocyte functions in these HLOs.

### Modeling DILI with diverse phenotypes using HLOs

**Fig. 2:**
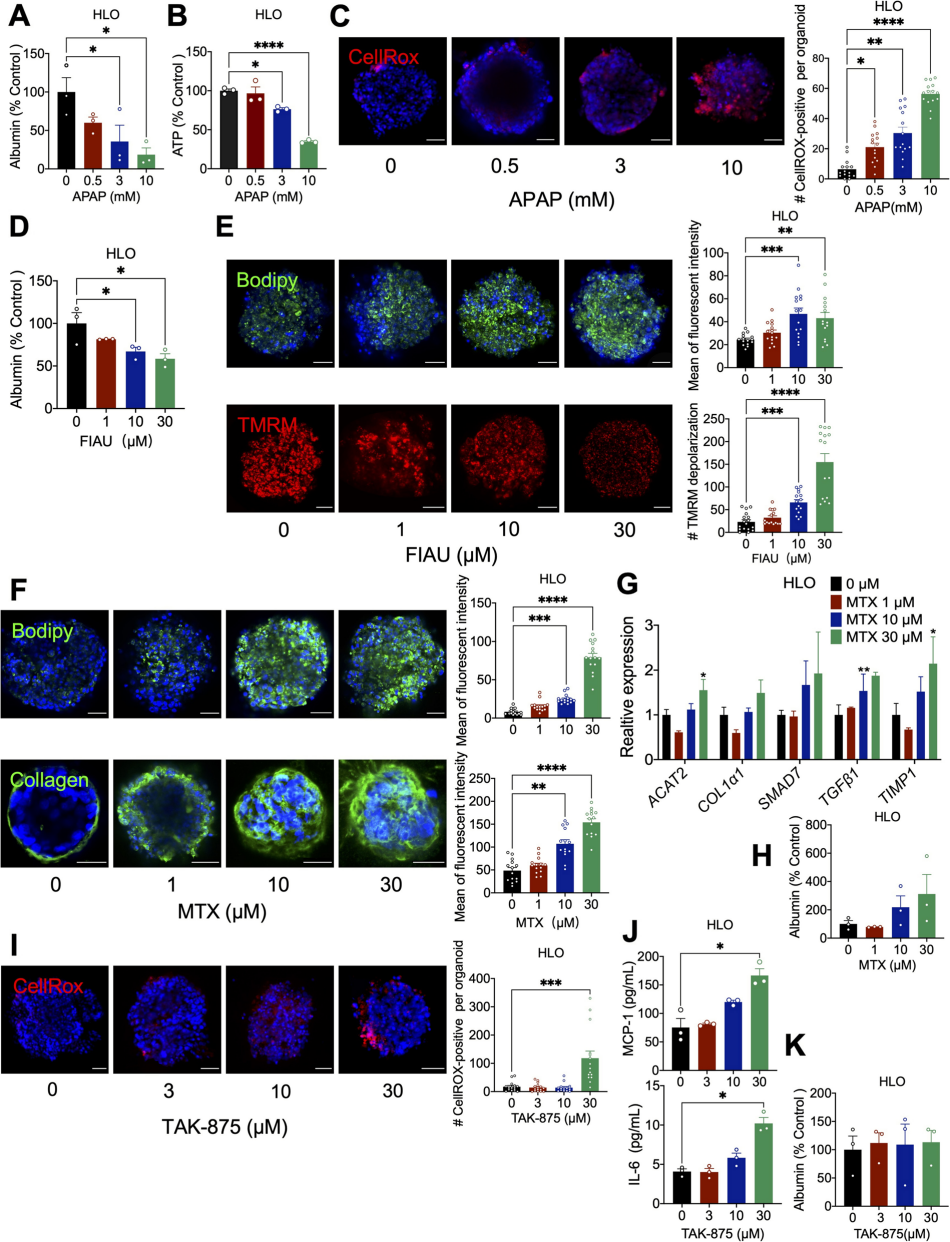
Modeling DILI with diverse phenotypes using HLOs. **A** Albumin secretion after administration of APAP at 0.5, 3, or 10 mM for 7 days in HLOs. *n* = 3, *P < 0.05. **B** ATP content analysis after administration of APAP at 0.5, 3, or 10 mM for 7 days in HLOs. *n* = 3, *P < 0.05. **C** Representative images of ROS intensity (CellROX in red, and DAPI in blue) after administration of APAP at 0.5, 3, or 10 mM for 7 days in HLOs. Scale bar, 50 μm. Right: Quantification of the number of CellROX-positive events per organoid. *n* = 15, *P < 0.05. **D** Albumin secretion after administration of FIAU at 1, 10, or 30 μM for 10 days. *n* = 3. **E** Representative images of lipid droplets (Bodipy in green, and DAPI in blue) and mitochondrial depolarization (TMRM in red) in the HLOs after administration of FIAU at 1, 10, or 30 μM for 10 days. Scale bars, 50 μm. Right: Quantification mean of Bodipy fluorescent intensity and quantification of the number of depolarization events per organoid. *n* = 15, *P < 0.05. **F** Representative images of lipid droplets (Bodipy in green, and DAPI in blue) and fibrosis (Collagen I in green, and DAPI in blue) in HLOs after administration of MTX at 1, 10, or 30 μM for 7 days. Scale bars, 50 μm. Right: Quantification mean of Bodipy and Collagen fluorescent intensity. *n* = 15, *P < 0.05. **G** Expression of fibrogenic marker genes in HLOs after treatment with MTX at 1, 10, or 30 μM for 7 days. *n* = 3, *P < 0.05. **H** Albumin secretion after MTX treatment for 7 days in HLOs. *n* = 3. **I** Representative images of ROS (CellROX in red, and DAPI in blue), after administration of TAK-875 at 3, 10 or 30 μM for 7 days in HLOs. Scale bars, 50 μm. Right: Quantifications of CellROX-positive events per organoid. *n* = 15. **J** Released MCP-1 and IL-6 in HLOs after 7 days of TAK-875 treatment. *n* = 3. *P < 0.05. **K** Albumin secretion after TAK-875 treatment for 7 days in HLOs. *n* = 3. Values represent means with SEM. *P* values were assessed by one-way ANOVA with Dunnett’s multiple comparisons test (**A**, **B**, **D**, **G**, **H**, **J**, **K**), Kruskal–Wallis tests (**C**, **E**, **F**, **I**)

We further assessed whether these HLOs can be used to model DILI with different toxins. HepG2, a liver hepatocellular carcinoma cell line, which was commonly adopted as an in vitro liver toxicity model, was applied here as control (Ramirez et al. 2018). Acetaminophen (APAP), an analgesic drug used to relieve chronic pain or to reduce fever, can directly cause injury to hepatocytes and produce DILI when overdosed (Yoon et al. 2016). A portion of APAP (~ 10%) is shunted by hepatic cytochrome CYP2E1 to oxidation, generating a highly reactive toxic metabolite N-acetyl-para-benzo-quinone imine (NAPQI). Excessive NAPQI depletes glutathione (GSH) and causes oxidative stress and mitochondrial dysfunction in hepatocytes, leading to the exhaustion of adenosine triphosphate (ATP) stores and liver injury. To evaluate the liver toxicity mediated by APAP, we treated both the HLOs and HepG2 spheroids with APAP at 0.5, 3, or 10 mM for 7 days. Treatment with APAP in HLOs resulted in a significant and dose-dependent decrease in albumin secretion and cellular ATP levels (Figs. 2A, B). The oxidative stress due to GSH depletion after APAP treatment was also demonstrated by a remarkable increase in the formation of reactive oxygen species (ROS), as revealed by the increased intensity of a fluorogenic probe, CellROX (Fig. 2C). Whereas in HepG2 spheroids, albumin level did not show clear change after APAP treatment, and a decrease in ATP level was only seen with high dose (10 mM) APAP treatment (Figs. S1A, B), although clear oxidative stress was also observed (Fig. S1C). These results highlight that HLOs were more sensitive to APAP-induced toxicity than HepG2 cells.

Fialuridine (FIAU), an antiviral nucleoside analog, caused specific liver steatosis and severe liver injury in humans (Jolly et al. 2020). Specifically, the FIAU-induced drug toxicity could not be well predicted using animal models, due to the species selective uptake of FIAU into mitochondria via the human equilibrative nucleoside transporter-1 (hENT-1, SLC29A1) (Lai et al. 2004). We then treated HLOs with FIAU to investigate whether HLOs can be adopted to predict FIAU-induced drug toxicities. FIAU was applied to HLOs and HepG2 spheroids at 1, 10, or 30 μM for 10 days at a daily basis. A dose-dependent decline in albumin secretion was noticed in HLOs after FIAU treatment, suggesting drug-induced liver cell injury (Fig. 2D). In line with previous discoveries in human clinical trials (Mckenzie et al. 1995), clear lipid accumulation was observed with Bodipy staining after FIAU treatment (Fig. 2E), suggesting liver steatosis induced by FIAU treatment. In addition, significant mitochondrial membrane depolarization, as indicated by decreased density of a mitochondrial membrane potential sensitive dye TMRM (Creed and McKenzie 2019), was also observed (Fig. 2E), which may contribute to the induction of steatosis by FIAU. In contrast, although being a human-originated cell line, FIAU treatment in HepG2 spheroids did not cause any effect on lipid accumulation or mitochondrial function (Figs. S1D, E), which is consistent with previous in vitro data (Jolly et al. 2020) in HepG2 and HepaRG models, suggesting the inability of these tumor cells in predicting FIAU-induced liver injury.

Methotrexate (MTX) is another drug that has been discovered to cause liver injury in humans associated with steatosis, and also fibrosis and even cirrhosis (Bath et al. 2014). We next determined whether HLOs can recapitulate these toxic effects, especially the fibrogenic effect, after MTX treatment. Indeed, HLOs treated with 1, 10, or 30 μM MTX for 7 days resulted in significant lipid accumulation and remarkable fibrosis, as evidenced by Collagen I staining and gene expression analysis of marker genes (Figs. 2F, G). Differently, MTX treatment did not cause clear lipid accumulation in HepG2 cells (Fig. S1F). Since the HepG2 spheroids did not contain stellate cells, which play a key role in the initiation and progression of liver fibrosis, no signal of Collagen I was detected in HepG2 cells after MTX treatment (Fig. S1F). In addition, no significant abnormalities in albumin secretion were observed in the HLOs or HepG2 cells after MTX treatment (Figs. 2H, S1G), consistent with the lack of predictive or diagnostic biomarkers for monitoring these toxicities in humans in clinic (Ezhilarasan 2021). Taken together, these results suggest that HLOs provide an available in vitro model for predicting drug-toxicity induced liver steatosis and fibrosis.

Idiosyncratic DILI (I-DILI) is a rare event that represents one of the difficult forms of liver toxicity to predict in the clinic. I-DILI is generally considered to be caused by the intrinsic chemical reactivity of drugs or reactive metabolites. Previous reports suggested that the immune-mediated responses, mitochondrial injury or bile salt export pump (BSEP) inhibition are involved in the development of I-DILI (Teschke 2019). We next tested whether the HLOs can be useful in predicting these types of responses using TAK-875 treatment. TAK-875 is a G protein-coupled receptor 40 (GPR40) agonist that was withdrawn from phase III clinical trials due to rare but serious incidents of DILI (Otieno et al. 2018). Subsequent studies have suggested that the oxidative stress, mitochondrial dysfunction, disrupted bile acid homeostasis, immune response, and certain genetic risk factors may all contribute to TAK-875-induced I-DILI (Mosedale et al. 2021). In HLOs after TAK-875 treatment, an increase in ROS formation was noticed after 1 week treatment at 30 μM (Fig. 2I), together with a significant increase in the release of inflammatory cytokines Monocyte chemoattractant protein-1 (MCP-1) and Interleukin 6 (IL-6) (Fig. 2J). No abnormalities in albumin secretion in HLOs after treatment was detected (Fig. 2K). These results demonstrated a detectable toxicity in oxidative stress and immune response with HLOs treated by TAK-875. HepG2 spheroids also showed accumulation of ROS after TAK-875 treatment (Fig. S1H), however, no effect in inflammatory cytokines was noticed (Fig. S1I), possibly due to the lack of immune cells.

### Predicting anti-hepatofibrotic drug efficacy based on high-content analysis using HLOs

**Fig. 3:**
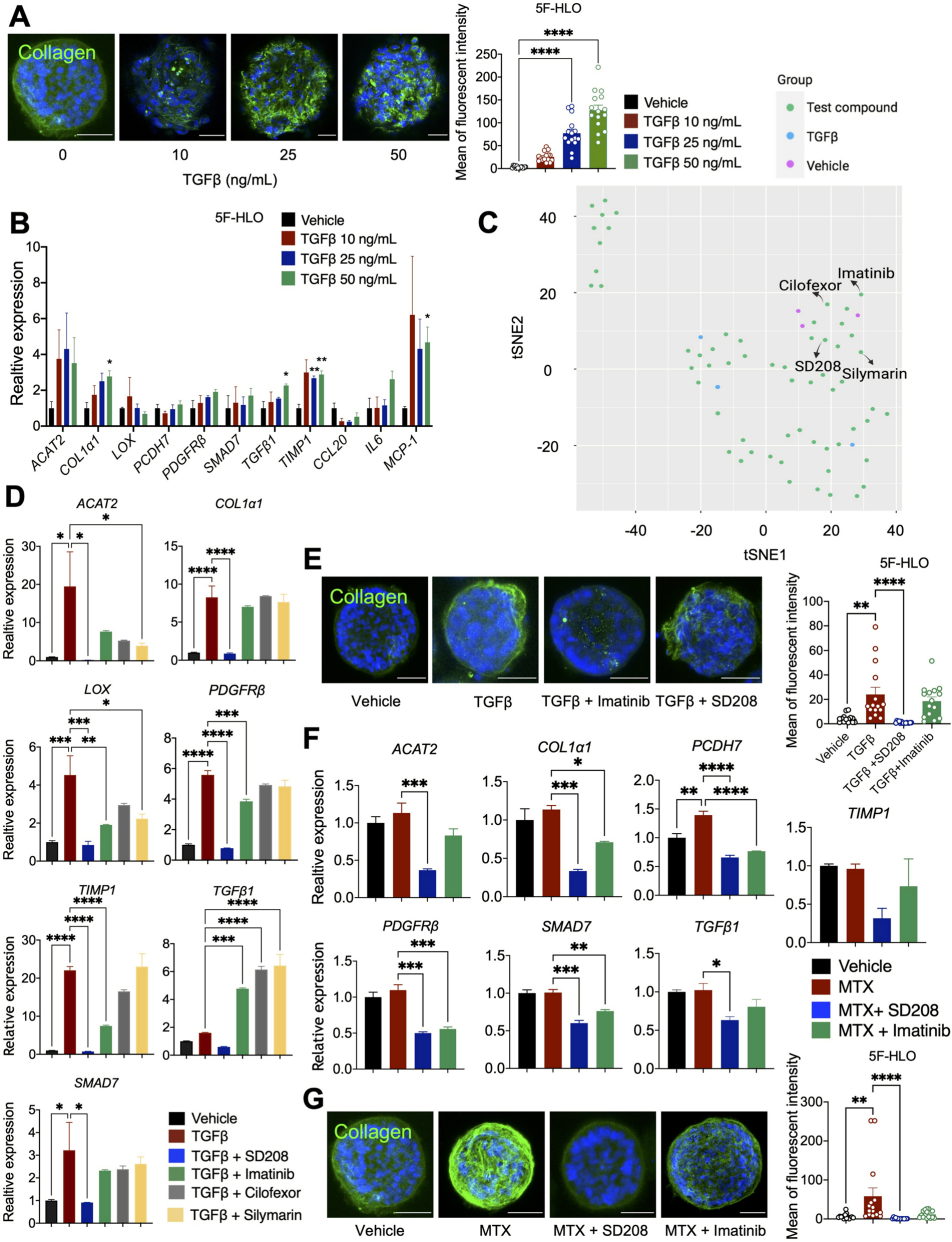
Predicting anti-hepatofibrotic drug efficacy based on high-content analysis using HLOs. **A** Representative images of fibrosis (Collagen I, green) in HLOs after administration of TGFβ1 at 10, 25, or 50 ng/mL for 3 days. Right: Quantification mean of Collagen fluorescent intensity. *n* = 15, *P < 0.05. **B** Gene expression analysis in HLOs after treatment with TGFβ1 at 10, 25, or 50 ng/mL for 3 days. *n* = 3, *P < 0.05. **C** tSNE analysis of potential anti-fibrotic effects of tested compounds in HLOs with HCA-based quantitative assessment screen. **D** Gene expression analysis in HLOs incubated with 25 ng/mL TGFβ and 1 μM anti-fibrotic compound (SD208, Imatinib, Cilofexor, or Silymarine) for 3 days. *n* = 3, *P < 0.05, versus TGFβ group. **E** HLOs were incubated with 25 ng/mL TGFβ and 1 μM anti-fibrotic compound (SD208, Imatinib) for 3 days. Scale bars, 50 μm. Representative images of fibrosis (Collagen I in green and DAPI in blue). Right: Quantification mean of fluorescent intensity. *n* = 15, *P < 0.05. **F** Gene expression analysis in HLOs incubated with 30 μM MTX and 1 μM SD208/ 4 μM Imatinib for 9 days. *n* = 3, *P < 0.05, versus MTX group. **G** HLOs were incubated with 30 μM MTX and 1 μM SD208/ 4 μM Imatinib for 9 days. Scale bars, 50 μm. Representative images of fibrosis (Collagen I in green and DAPI in blue). Right: Quantification mean of fluorescent intensity. *n* = 15, *P < 0.05. Values represent means with SEM. *P* values were assessed by one-way ANOVA with Dunnett’s multiple comparisons test (**B**, **D**, **F**), and Kruskal–Wallis tests (**A**, **E**, **G**)

With the above-mentioned experimental settings, we have demonstrated that HLOs were able to model diverse toxic phenotypes induced by different drugs, such as steatosis, fibrosis, mitochondrial dysfunction, oxidative stress and immune response. We next wanted to examine whether these HLOs can be used to predict drug toxicity or efficacy in a high-throughput manner. Liver fibrosis is a wound-healing response generated against an insult that causes liver injury, which is seen in many disease conditions, including DILI and NASH (Ezhilarasan 2021; Loomba et al. 2021). Liver fibrosis can progress to liver cirrhosis, leading to liver cancer or liver failure, with no effective treatment so far (Maharjan et al. 2021). We thus wanted to establish a high-throughput platform with HLOs for anti-fibrogenic drug screening. Transforming growth factor beta (TGFβ) and lipopolysaccharides (LPS) are well-known common profibrogenic factors that induce liver fibrosis in many liver diseases (Kisseleva and Brenner 2021). Indeed, administration of TGFβ at 10, 25, or 50 ng/mL or LPS at 100 ng/mL or 200 ng/mL for 3 days all resulted in increased Collagen I deposition (Figs. 3A, S2A). Gene analysis demonstrated more enhanced pro-fibrogenic effects after TGFβ treatment when compared to LPS treatment (Figs. 3B, S2B). In addition, massive fragmentation was observed in Collagen I staining after treatment with TGFβ at 50 ng/mL or LPS at 200 ng/mL, possibly due to cell death. We thus adopted TGFβ treatment at 25 ng/mL as a fibrogenic model for later screening.

The staining signal for the fibrotic marker Collagen I was used as a readout for liver fibrosis, and the high-content analysis (HCA) was performed to assess the possible anti-fibrogenic effects of 60 compounds (Table S1). These compounds were chosen for their potentiality to inhibit fibrosis based on literature search (Kisseleva and Brenner 2021; Zheng et al. 2011) (Table S2). Vehicle-treated cells were adopted as control. High-content analysis was then setup in which a total of 16 features in three types of organoids relevant to organoid shape, collagen distribution and fluorescence intensity were extracted and quantified to assess the anti-fibrotic efficacies (Fig. S2C). T-distributed stochastic neighbor embedding (tSNE) analysis was then performed to identify major clusters among compounds (Supplementary material 1). Interestingly, ten compounds were grouped together with three vehicle groups, suggesting a possible anti-fibrogenic effect with these compounds, in which SD208 is an identified TGFβ inhibitor (Fig. 3C).

We further went on to validate 4 of these compounds (SD208, Imatinib, Cilofexor, Silymarin) in suppressing fibrosis induced by TGFβ treatment. Gene expression analysis demonstrated that SD208 and Imatinib treatment suppressed the expression of several fibrogenic markers, e.g. *ACAT2, COL1α1, LOX, SMAD7, TGFβ1, TIMP1*, with a higher efficacy of SD208; whereas Cilofexor or Silymarin did not show a significant effect (Fig. 3D). Staining of Collagen I confirmed the anti-hepatofibrotic effect of SD208 in HLOs with TGFβ treatment (Fig. 3E). Further analysis with MTX induced- and LPS induced-liver fibrotic HLO models also demonstrated potential anti-fibrogenic effects of SD208 in both MTX and LPS-induced liver fibrosis, and Imatinib in MTX-induced fibrosis (Figs. 3F, G; S2D, E).

## Discussion

In this study, we first demonstrated the utility of HLOs in recapitulating diverse phenotypes of DILI by applying four different tool compounds—APAP, FIAU, MTX and TAK-875, each of which causes liver injury under different mechanisms. Different toxic-relevant phenotypes were observed mostly in accordance with previous human data, including steatosis, fibrosis, and immune responses, proving that HLOs can be applied for safety testing. Compared to many other in vitro toxicity-predicting cellular systems with only hepatocytes, the major advantage of HLOs is the existence of multiple functional cell types involved in DILI, which therefore enables the detection of fibrosis, immune responses, and cholestasis, all contributing heavily to DILI. In addition, the comparison of HLOs with HepG2 also suggested that the hepatocytes in HLOs were more sensitive in detecting drug-induced injuries than HepG2 tumor cells. Although more tests with thorough phenotypic studies and different compounds are needed before we learn all the pros and cons of HLOs, the application of HLOs in drug safety testing is promising.

One of the limitations of this study is that we used the liver hepatocellular carcinoma HepG2 cells as control. Although HepG2 was commonly adopted as an in vitro liver toxicity model (Ramirez et al. 2018), it is hard to accurately reflect the physiological response as a tumor cell line. In comparison, human primary hepatocytes can be a better control here. However, human primary hepatocytes are hard to obtain and can lose their hepatic characteristics quickly in vitro. Significant advances have been achieved in the development of in vitro models using human primary hepatocytes that can predict DILI, such as the 3D spheroid model that can maintain metabolic competence for prolonged durations (Huch et al. 2015; Xiang et al. 2019; Zhang et al. 2018a). Considering that the non-parenchymal cells (NPCs) fractions in liver are major drivers of liver fibrosis progress or immune responses in response to toxins, for example HSCs (Zhang et al. 2016), and Kupffer cells (Damm et al. 2013), many attempts have also been executed aiming to recreate a more physiological cell model by co-culturing primary liver non-parenchymal cells with human primary hepatocytes. For example, co-cultures of hepatocytes with either Kupffer cells, HSCs, or biliary cells have also been shown to increase the predictive power of cell culture models (Baze et al. 2018; Feaver et al. 2016). While incorporating NPC fractions is a viable option, the use of a mixture cannot be controlled and induced 3D co-culture constructs lack cytoarchitecture and physiologically relevant tissue-tissue interfaces that have been demonstrated to be a key driver of cell function (Bale et al. 2016; Jang et al. 2019). Nevertheless, direct functional comparisons with HLO models derived from hPSCs used in this study and 3D models with human primary hepatocytes and other NPC fractions are warranted in the future.

In the second part of this manuscript, we initiated an effort in devising a high-throughput platform using HLOs for anti-fibrogenic drug screening. Sixteen features with high-content analysis using Collagen I staining as the main readout were extracted and used for efficacy calculating. Sixty compounds were chosen for testing, in which 10 compounds showed potential anti-fibrogenic efficacy from the screening, and 2 of 4 compounds were successfully validated later. Different treatments to establish fibrosis models and more parameters to reflect the anti-fibrogenic effects will surely improve the sensitivity and robustness of this system. Nonetheless, SD208 was screened out that could significantly reduce fibrosis induced by treatment with TGFβ, MTX or LPS, highlighting the potentiality of this system as a simple, convenient, and effective anti-fibrosis drug screening platform.

It is currently acknowledged that Kupffer cell-derived TGFβ1 induces HSCs activation and collagen production (Sato et al. 2016). TGFβ1 is also considered as the key cytokine that drives liver fibrosis in patients with chronic liver disease (Weng et al. 2009). The in vitro studies have also shown that Kupffer cells can induce the expression of platelet-derived growth factor (PDGF) on HSCs, thus enhancing HSCs proliferation in response to PDGF (Sato et al. 2016). In addition, IL-6 and MCP-1, which are produced by the activated Kupffer cells, are also mitogenic and chemoattractant for HSCs (Gregory et al. 1998; Wang et al. 2017). In this regard, SD208 and Imatinib, may exert their anti-fibrotic effects through inhibition of TGFβ1 and PDGF receptor in Kupffer cells in HLOs, respectively. Further investigation of whether SD208 or Imatinib could be used in the therapy of liver fibrosis and the detailed mechanism combined with liver fibrosis model or even humanized mice could offer more insights. For example, we could transplant human liver organoids into immunodeficient animals and induce liver fibrosis with CCL_4_ or other toxins to establish in vivo human liver fibrosis models for drug tests (Benten et al. 2018; Ma et al. 2022; Wang et al. 2022).

## Conclusions

In summary, we have demonstrated the utility of HLOs in modeling DILI *in dish* and in high-throughput anti-fibrogenesis compound screening in this study. However, despite of the functional responses of HLOs to drugs that induce fibrosis or immune responses, suggesting functional stellate and immune cells in HLOs, we did not provide careful studies of the cell compositions as well as the functions of each cell type in HLOs in this study. Besides, the hepatocytes in HLOs also retain a fetal-like stage, as revealed by relatively low albumin secretion. Efforts with systematic characterization of each cell type in HLOs, incorporation of developmental findings and bioengineer approaches to improve the differentiation of HLOs, and comparisons between HLOs and in vivo liver responses under different conditions will certainly be helpful to accelerate the applications of HLOs in clinical and pre-clinical studies.

## Methods

### Generation of human liver organoids (HLOs) and HepG2 3D spheroids

The 1016 human induced pluripotent stem cells (hiPSCs) clone used in this study was kindly provided by the department of stem cell and regenerative biology in Harvard University and Harvard stem cell institute. The hiPSCs were differentiated into foregut using a previously described method (Ouchi et al. 2019). In brief, hiPSCs were detached by Accutase (Thermo Fisher Scientific) and were seeded on Gelterx (Gibco, A1413202) coated tissue culture plates with 100,000 cells/cm^2^. The medium was changed to RPMI 1640 (Gibco, C11875500CP) with 100 ng/mL activin A (Peprotech, 120-14E) and 50 ng/mL bone morphogenetic protein 4 (BMP4; Peprotech, 120–05) at day 1, 100 ng/mL activin A and 0.2% fetal bovine serum (FBS; Gibco, 16000-044) at day 2, and 100 ng/mL activin A and 2% FBS at day 3. On day 4 to 6, cells were cultured in Advanced DMEM/F12 (Gibco, 12634028) with 1 × B27 (Gibco, 12587010), 1 × N2 (Gibco, 17502048), 1 × GlutaMaX (Gibco, 35050-061), 500 ng/mL FGF4 (Peprotech, AF-100-31), 3 μM CHIR99021 (Sigma, SML1046) and 1% penicillin/streptomycin (Beyotime, C0222). Cells were maintained at 37 ℃ in humidified air with 5% CO_2_, and the medium was replaced every day.

For the HLO induction, at day 6 the foregut cells were detached easily by gentle pipetting. The foregut cells were then centrifuged at 800 rpm for 2 min. Cells were suspended in Matrigel (Corning, 356237). A total of 300,000 cells were embedded in 50 μL Matrigel drop on the dishes in RA medium [Advanced DMEM/F12 with 1 × B27, 1 × N2, 1 × GlutaMaX, 2 μM retinoic acid (RA; Sigma, R2625), and 1% penicillin/streptomycin] and cultured for 4 days. During day 10–20, the media was switched to liver maturation medium [Hepatocyte Culture Medium (HCM; Lonza, CC-3198) prepared as manufacturer’s instructions, except no EGF, and supplemented with 10 ng/mL hepatocyte growth factor (HGF; PeproTech, 100-39), 0.1 μM Dexamethasone (Dex; Sigma, D4902), 20 ng/mL Oncostatin M (OSM; Peprotech, 300-10) and 1% penicillin/streptomycin]. Cells were maintained at 37 ℃ in humidified air with 5% CO_2_, and the medium was replaced every 3 days (Thompson and Takebe 2020).

Alternatively, at day 6 the foregut cells were detached by accutase, and then centrifuged at 800 rpm for 2 min. A total of 100,000 cells were embedded in 50 μL Matrigel drop on the dishes in 5F medium [Advanced DMEM/F12 with 1 × B27, 1 × N2, 1 × GlutaMaX, 5 ng/mL FGF2 (Peprotech, 100-18B), 10 ng/mL VEGF (Peprotech, 100–20-10), 20 ng/mL EGF (Peprotech, AF-100–15), 3 μM CHIR99021, 0.5 μM A83-01 (Tocris, 2939), 50 μg/mL ascorbic acid (Sigma, A5960) and 1% penicillin/streptomycin] for 4 days (Zhang et al. 2018b). After 4 days’ treatment, the media was switched to RA media for another 4 days. At day 15, organoids were harvested from Matrigel by scratching and pipetting and re-embedded in Matrigel on the ultra-low attachment multiwall plate (Corning, 3471) in liver maturation media for 10 days. Cells were maintained at 37 ℃ in humidified air with 5% CO_2_, and the medium was added every 2 days (Shinozawa et al. 2021).

HepG2 cells provided by Cell Bank (Type Culture Collection Committee, Chinese Academy of Sciences) were resuscitated in Dulbecco’s modified Eagle’s medium (DMEM; Gibco, C11995500CP) supplemented with 10% FBS and 1% penicillin/ streptomycin and seeded in 6 well plate at 2 × 10^4^ cells/cm^2^. Cells were maintained at 37 ℃ in humidified air with 5% CO_2_, and the medium was replaced every day, and passaged every 2 days. For 3D HepG2 spheroids formation, cells were seeded at 8 × 10^4^ cells/well in a 96-well plate (Corning, 4442). Cells were maintained in DMEM containing 10% FBS and 1% penicillin/ streptomycin in a humidified incubator with 5% CO_2_ at 37 °C for 2 days before treatment with drugs.

### RNA isolation and RT-qPCR

Total RNA was isolated from cells by Trizol reagent (Thermo Fisher Scientific, 10296010) according to the manufacturer’s instructions. One microgram RNA was reverse transcribed into cDNA with PrimeScript reverse transcription kit (Takara, RR047A). Quantitative real-time PCR was carried out using SYBR Green supermix (Applied Biosystems, 4472908). The sequences of primers used are as follows:

- *ALB*-F: GCCTTTGCTCAGTATCTT
- *ALB*-R: AGGTTTGGGTTGTCATCT
- *CYP3A4*-F: TTCAGCAAGAAGAACAAGGACAA
- *CYP3A4*-R: GGTTGAAGAAGTCCTCCTAAGC
- *CYP2B6*-F: GCACTCCTCACAGGACTCTTG
- *CYP2B6*-R: CCCAGGTGTACCGTGAAGAC
- *CYP2E1*-F: ATGTCTGCCCTCGGAGTCA
- *CYP2E1*-R: CGATGATGGGAAGCGGGAAA
- *PDGFRβ*-F: AGCACCTTCGTTCTGACCTG
- *PDGFRβ*-R: TATTCTCCCGTGTCTAGCCCA
- *COL1α1*-F: GAGGGCCAAGACGAAGACATC
- *COL1α1*-R: CAGATCACGTCATCGCACAAC
- *KRT19*-F: AACGGCGAGCTAGAGGTGA
- *KRT19*-R: GGATGGTCGTGTAGTAGTGGC
- *SOX9*-F: AGCGAACGCACATCAAGAC
- *SOX9*-R: CTGTAGGCGATCTGTTGGGG
- *CD14*-F: TCTCTGTCCCCACAAGTTCC
- *CD14*-R: CCCGTCCAGTGTCAGGTTATC
- *CD68*-F: GAACCCCAACAAAACCAAG
- *CD68*-R: GATGAGAGGCAGCAAGATG
- *ACTA2*-F: AAAAGACAGCTACGTGGGTGA
- *ACTA2*-R: GCCATGTTCTATCGGGTACTTC
- *PCDH7*-F: GGATCGGGTGAGGTGACTTTC
- *PCDH7*-R: GTTCTCGTCGAAGATCATCTGAC
- *LOX*-F: CGGCGGAGGAAAACTGTCT
- *LOX*-R: TCGGCTGGGTAAGAAATCTGA
- *SMAD7*-F: TTCCTCCGCTGAAACAGGG
- *SMAD7*-R: CCTCCCAGTATGCCACCAC
- *TGFβ1*-F: GGCCAGATCCTGTCCAAGC
- *TGFβ1*-R: GTGGGTTTCCACCATTAGCAC
- *TIMP1*-F: CTTCTGCAATTCCGACCTCGT
- *TIMP1*-R: ACGCTGGTATAAGGTGGTCTG
- *CCL20*-F: TGCTGTACCAAGAGTTTGCTC
- *CCL20*-R: CGCACACAGACAACTTTTTCTTT
- *IL6*-F: ACTCACCTCTTCAGAACGAATTG
- *IL6*-R: CCATCTTTGGAAGGTTCAGGTTG
- *MCP-1*-F: CAGCCAGATGCAATCAATGCC
- *MCP-1*-R: TGGAATCCTGAACCCACTTCT
- *GAPDH*-F: GGAGCGAGATCCCTCCAAAAT
- *GAPDH*-R: GGCTGTTGTCATACTTCTCATGG

### ELISA

Supernatants from differentiated HLOs or HepG2 spheroids were collected at the end of differentiation or after drug treatment. Levels of human albumin, MCP-1, and IL-6 were measured by the human serum albumin duoset ELISA (R&D system, DY1455), human MCP-1 (CCL2) mini TMB ELISA development kit (Peprotech, 900-TM31), and human IL-6 mini TMB ELISA development kit (Peprotech, 900-TM16), respectively.

### Immunofluorescence staining

Organoids were fixed with 4% paraformaldehyde (PFA) in PBS (30 min, room temperature), followed by permeabilization in 0.2% Triton X-100 in PBS (1 h, room temperature) and blocking (1% BSA in PBS) for 1 h. Samples were then incubated with primary antibodies against Albumin (1:100, R&D systems, MAB1455), SOX9 (1:500, Millipore, AB5535), PDGFRβ (1:200, Abcam, ab32570), Collagen I (1:500, Abcam, ab34710) at 4 ℃ for 24 h and secondary antibodies Alexa 488 donkey-anti-rabbit (1:200, Invitrogen, R37118), Alexa 647 donkey-anti-mouse (1:1000, Invitrogen, A21235) for 3 h. Nuclei were counterstained with 4’,6-diamidino-2-phenylindole (DAPI) (1:2000, Beyotime, C1002). Organoids were washed with PBS between different steps. Stained organoids were imaged by confocal microscope (Zeiss LSM 880) (Dekkers et al. 2019). All image analysis was performed using the ImageJ-Fiji software.

### CDCFDA uptake and release

To visualize bile canaliculi and MRP2 activity, cells were incubated with the HCM containing 2 μM CDCFDA (Meilunbio, MB6180) for 30 min at 37 °C. The samples were then washed twice with pre-warmed PBS. The green fluorescence of CDCF (CDCF is the fluorescent metabolite of CDCFDA, which can be secreted by MRP2 and accumulated in canaliculus-like structures (Torok et al. 2020) was visualized by confocal microscopy (Zeiss LSM 880). The organoids were subsequently washed with PBS and refilled with liver maturation medium for 72 h before images of the CDCFDA release were captured using a confocal microscope (Zeiss LSM 880).

### PAS staining

To assess glycogen storage, the organoids were stained by a PAS staining kit (Beyotime, C0142S), according to the manufacturer’s protocol. Then samples were imaged by microscope (Revolve 6).

### LDL uptake

LDL uptake was detected with DiI-LDL (Yeasen, 20614ES76). The organoids were treated with 40 µg/ mL DiI-LDL for 2 h, then the organoids were collected, fixed, incubated with DAPI and imaged by confocal microscope as mentioned above.

### Drug treatment

About 100 HLOs per well were incubated with a culture medium containing 5% Matrigel and acetaminophen (APAP; MCE, T0065), methotrexate (MTX; Selleck, S1210), fasiglifam (TAK-875; MCE, 1000413-72-8) for 7 days, fialuridine (FIAU; Sigma, SML0632) for 10 days, or TGFβ1 (Peprotech, AF-100-21C) /LPS (Sigma, L4391) for 3 days, in 96 well ultra-low attachment multiwell plate (Corning, 3473), respectively.

### TMRM/ CellROX/ Bodipy staining

After treatment with drugs, organoids were collected and stained with CellROX (Thermo Fisher, C10422) to visualize cellular oxidative stress, tetramethylrhodamine methyl ester (TMRM; Thermo Fisher, T668) to visualize active mitochondria, and 4,4-difluoro-1,3,5,7,8-pentamethyl-4-bora-3a,4a-diaza-s-indacene (BODIPY 493/503; GLPBIO, GC42959) to visualize lipid droplet accumulation (Qiu and Simon 2016), respectively. Staining solution was prepared in a blank medium (HCM for HLO TMRM/ CellROX staining, DMEM for HepG2 TMRM/ CellROX staining, PBS for BODIPY staining), and organoids were kept in staining solution for 15 min at 37 °C. The TMRM stained organoids were imaged immediately. The CellROX or BODIPY stained organoids were collected, fixed, incubated with DAPI and imaged by the confocal microscope as mentioned above. Using ImageJ-Fiji software, the fluorescent images of z-stack were combined into one image, followed by fluorescence quantification (Creed and McKenzie 2019; Ouchi et al. 2019). All of the plots were generated using Prism 9 software (GraphPad).

### Compounds preparation

The small molecule compound library for screening were purchased from Topscience with details listed in Tables S1 and S2. Stock solution of each compound was prepared by dissolving the compound in dimethyl sulfoxide (DMSO; Yeasen) at a concentration of 10 mM.

### High-content imaging and analysis

About 100 organoids per well were incubated with culture medium containing 5% Matrigel, TGFβ1 (25 ng/mL) and the compound (1 μM or 0.1 μM of each compound) for 3 days in the 96 well high content imaging plate (Corning, 4580) before staining for Collagen I as mentioned above. Organoids treated only with DMSO were used as vehicle group, and treated with DMSO and TGFβ1 (25 ng/mL) as TGF β group. The images were acquired using a high content screening system (Cellomics ArrayScan VTI, Thermo Fisher Scientific). All images were taken with a 5 × air objective. Nine high-resolution images (1024 × 1024 pixels, 5 z-stack images compressed into 1) were taken per well. Image segmentation and feature extraction were performed with Cellomics HCS studio 3.0 image analysis system, in which individual organoids were classified into 3 types and 16 features describing organoid shape, collagen distribution and fluorescence intensity were extracted from 1-channel (DAPI) or 2-channel (Collagen I), respectively. T-distributed stochastic neighbor embedding (tSNE) analysis was then used to generate clusters to distinguish compounds with potential anti-fibrosis efficacy (Supplementary material 1).

### Statistics

The unpaired, two-tailed Student’s *t* test was used for experiments with two groups and one-way ANOVA Dunnett’s multiple comparisons tests were used for parametric data, and the Kruskal–Wallis tests was used for nonparametric data that contained more than two groups. All statistical analyses were performed using Prism 9 (GraphPad). Error bars in graphs are defined in the figure legends and represent the mean with SEM.

## Supplementary Information

**Additional file 1: Figure S1.** Phenotypic analysis using HepG2 spheroids after drug treatment. **Figure S2.** Predicting anti-hepatofibrotic drug efficacy based on high-content analysis using HLOs. **Table S1.** List of all 60 test compounds and CAS numbers in HCA screening. **Table S2.** List of all 60 test compounds detail information.

## Data Availability

All supporting data and materials are available within the article and its additional files.

## Abbreviations

hiPSCs: Human induced pluripotent stem cells
HLO: Human liver organoid
DILI: Drug-induced liver injury
CYP: Cytochrome P450
NASH: Non-alcoholic steatohepatitis
AIH: Autoimmune hepatitis
HLOs: Human liver organoids
HCA: High-content analysis
RA: Retinoid acid
FGF2: Fibroblast growth factor 2
VEGF: Vascular endothelial growth factor
EGF: Epidermal growth factor
MRP2: Multi-drug resistance protein 2
PAS: Periodic acid-Schiff
APAP: Acetaminophen
NAPQI: N-acetyl-para-benzo-quinone imine
GSH: Glutathione
ATP: Adenosine triphosphate
ROS: Reactive oxygen species
FIAU: Fialuridine
hENT-1: Human equilibrative nucleoside transporter-1
MTX: Methotrexate
I-DILI: Idiosyncratic DILI
BSEP: Bile salt export pump
GPR40: G protein-coupled receptor 40
MCP-1: Monocyte chemoattractant protein-1
IL-6: Interleukin 6
TGFβ: Transforming growth factor beta
LPS: Lipopolysaccharides
tSNE: T-distributed stochastic neighbor embedding
NPCs: Non-parenchymal cells
BMP4: Bone morphogenetic protein 4
FBS: Fetal bovine serum
HGF: Hepatocyte growth factor
PFA: Paraformaldehyde
DAPI: 4’,6-Diamidino-2-phenylindole
TMRM: Tetramethylrhodamine methyl ester
BODIPY 493/503: 4,4-Difluoro-1,3,5,7,8-pentamethyl-4-bora-3a,4a-diaza-s-indacene
