# Supplementary material for: Modeling drug-induced liver injury and screening for anti-hepatofibrotic compounds using human PSC-derived organoids
Source: Cell Regen. 2023 Mar 3;12:6. doi: 10.1186/s13619-022-00148-1 (PMC9981852; doi:10.1186/s13619-022-00148-1)
Supplement: Supplementary file 1 — Additional file 1: Figure S1. Phenotypic analysis using HepG2 spheroids after drug treatment. Figure S2. Predicting anti-hepatofibrotic drug efficacy based on high-content analysis using HLOs. Table S1. List of all 60 test compounds and CAS numbers in HCA screening. Table S2. List of all 60 test compounds detail information. [file 13619_2022_148_MOESM1_ESM.zip › Xiaoshan HLO Supplemental Information-revisedR2.docx]

**Supplementary Figures and Figure legends**


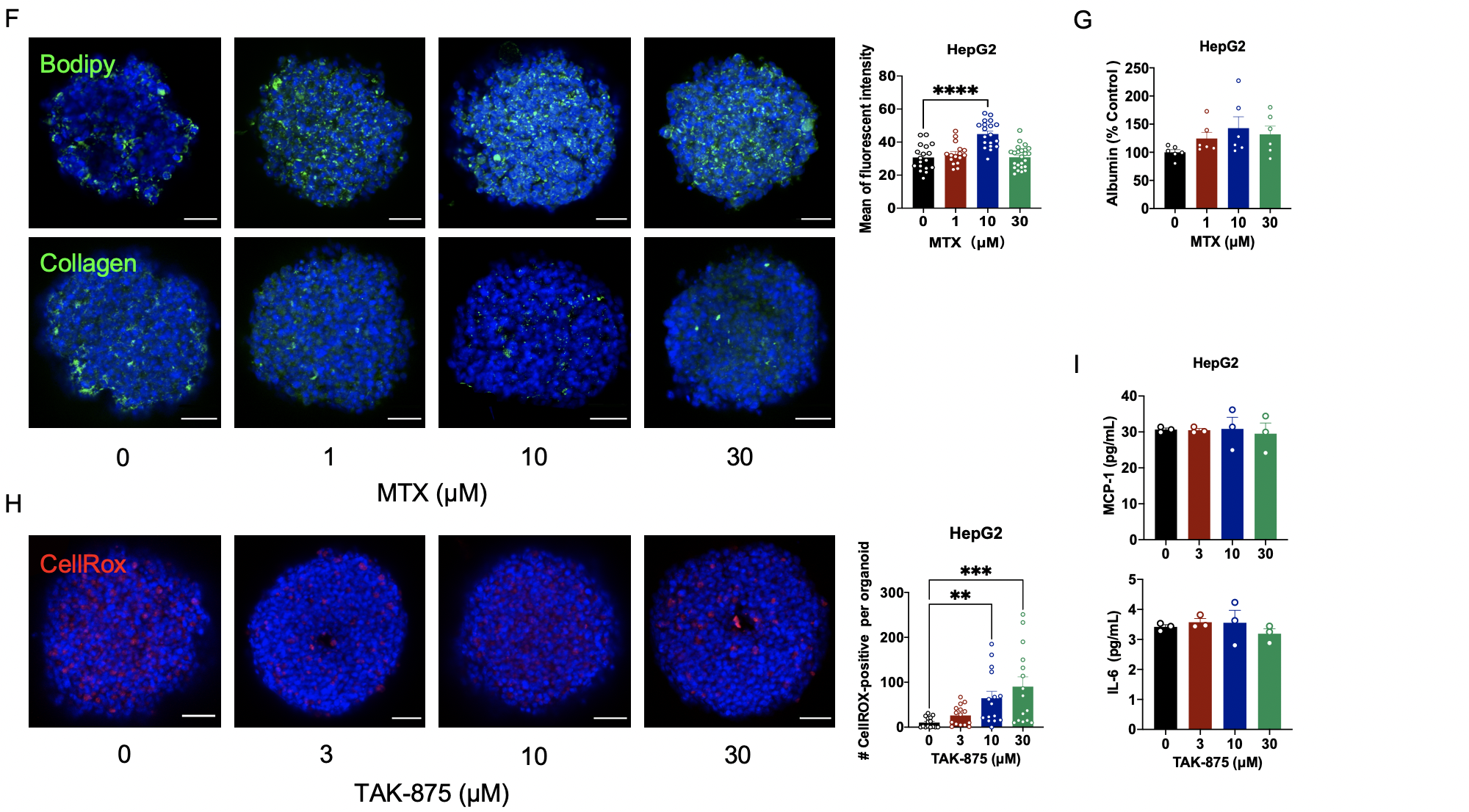

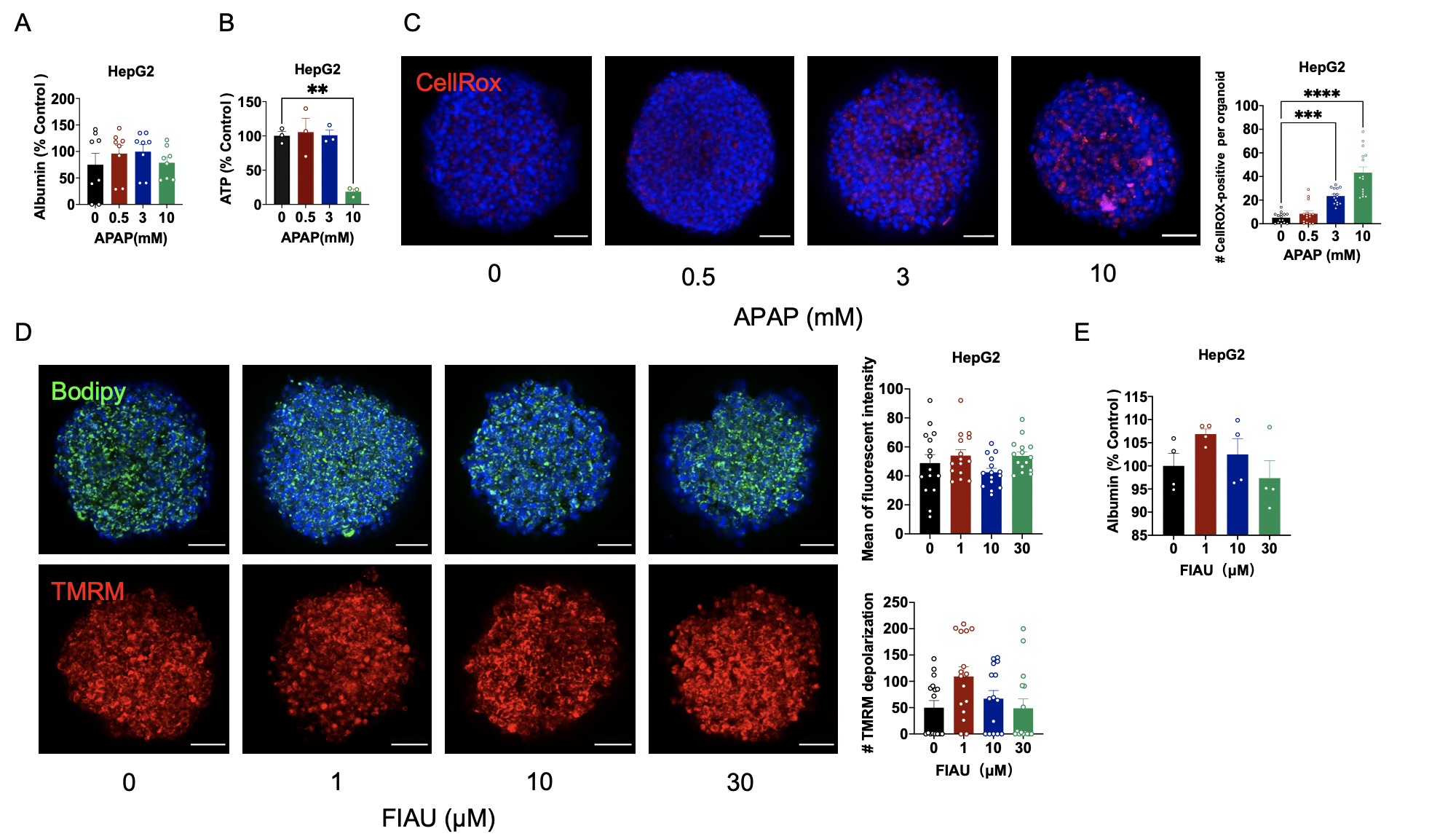


**Figure S1. Phenotypic analysis using HepG2 spheroids after drug treatment. A** Albumin secretion analysis in HepG2 spheroids after APAP treatment for 7 days. n = 3. **B** ATP content analysis after administration of APAP at 0.5, 3, or 10 mM for 7 days in HepG2 spheroids. n = 3. **P* < 0.05. **C** Representative images of ROS intensity (CellROX in red, and DAPI in blue) after administration of APAP at 0.5, 3, or 10 mM for 7 days in HepG2 spheroids. Scale bar, 50 μm. Right: Quantification of the number of CellROX-positive events per organoid. n = 15, **P* < 0.05. **D** Representative images of lipid droplets (Bodipy in green, and DAPI in blue) and mitochondrial depolarization (TMRM in red) in HepG2 spheroids after administration of FIAU at 1, 10, or 30 μM for 10 days. Scale bars, 50 μm. Right: Quantification mean of Bodipy fluorescent intensity and the number of depolarization events per organoid. n = 15. **E** Albumin secretion after FIAU treatment for 10 days in HepG2 spheroids. n = 3. **F** Representative images of lipid droplets (Bodipy in green, and DAPI in blue) and fibrosis (Collagen I in green, and DAPI in blue) in HepG2 spheroids after administration of MTX at 1, 10, or 30 μM for 7 days. Scale bars, 50 μm. Right: Quantification mean of Bodipy and Collagen fluorescent intensity. n = 15, * *P* < 0.05. **G** Albumin secretion analysis after MTX treatment for 7 days in HepG2 spheroids. n = 6. **H** Representative images of ROS (CellROX in red, and DAPI in blue), after administration of TAK-875 at 3, 10 or 30 μM for 7 days in HepG2 spheroids. Scale bars, 50 μm. Right: Quantifications of CellROX-positive events per organoid. n = 15, * *P* < 0.05. **I** MCP-1 and IL-6 analysis in HepG2 spheroids after 7 days of TAK-875 treatment. n = 3. Values represent means with SEM. *P* values were assessed by one-way ANOVA with Dunnett’s multiple comparisons test (A, B, E, G, I), and Kruskal-Wallis tests (C, D, F, H).


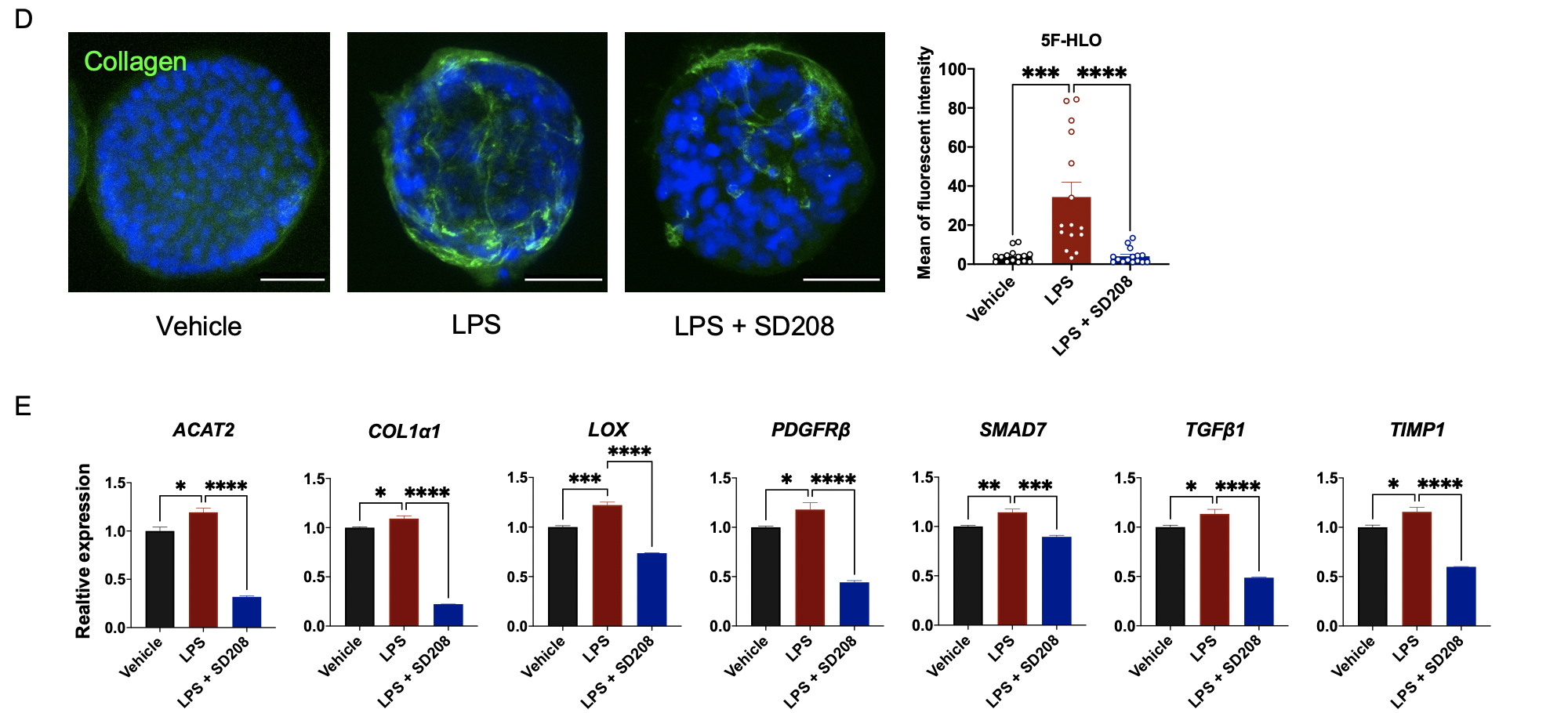

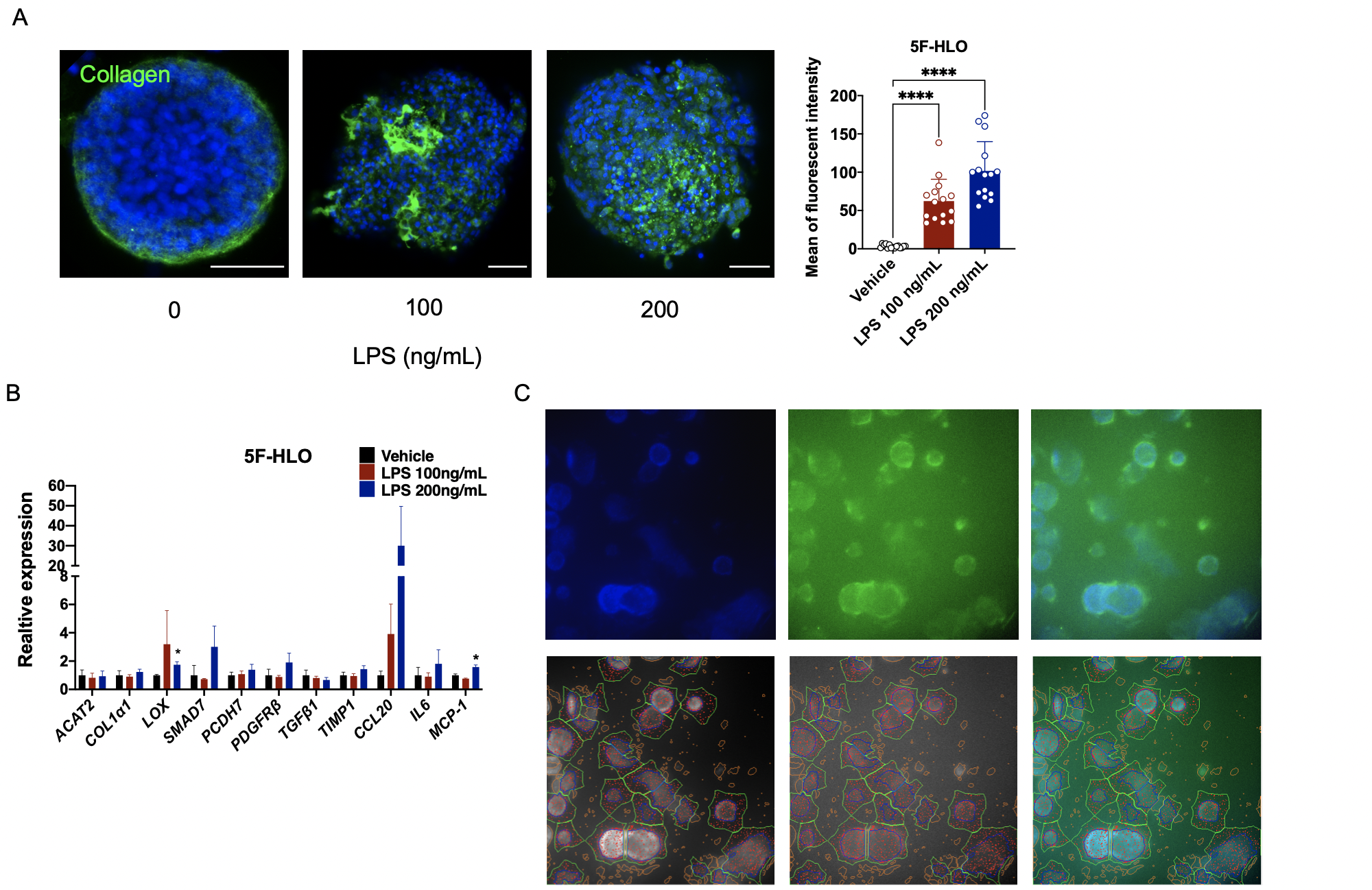


**Figure S2. Predicting anti-hepatofibrotic drug efficacy based on high-content analysis using HLOs. A** Representative images of fibrosis (Collagen in green, and DAPI in blue) in HLOs after administration of LPS at 100 or 200 ng/mL for 3 days. Scale bar, 50 μm. Right: Quantification mean of Collagen fluorescent intensity. n = 15, **P* < 0.05. **B** Genes expression analysis in HLOs after treatment with LPS at 100 or 200 ng/mL for 3 days. n = 3, **P* < 0.05. **C** Image segmentation and feature extraction were performed by Cellomics HCS Studio 3.0. Images of organoid were classified into 3 types and 5~6 features were extracted from each type for quantitative analysis. **D** Representative images of fibrosis (Collagen I in green, and DAPI in blue) in HLOs after treatment with 100 ng/mL LPS and 1 μM SD208 for 3 days. Scale bar, 50 μm. **E** Gene expression analysis in HLOs after treatment with 100 ng/mL LPS and 1 μM SD208 for 3 days. n = 3, **P* < 0.05, versus LPS group. Right: Quantification mean of fluorescent intensity. **P* < 0.05. Values represent means with SEM. *P* values were assessed by one-way ANOVA with Dunnett’s multiple comparisons test (B, E), and Kruskal-Wallis tests (A, D).
