# Supplementary material for: Modeling drug-induced liver injury and screening for anti-hepatofibrotic compounds using human PSC-derived organoids
Source: Cell Regen. 2023 Mar 3;12:6. doi: 10.1186/s13619-022-00148-1 (PMC9981852; doi:10.1186/s13619-022-00148-1)
Supplement: Supplementary file 1 — Additional file 1: Figure S1. Phenotypic analysis using HepG2 spheroids after drug treatment. Figure S2. Predicting anti-hepatofibrotic drug efficacy based on high-content analysis using HLOs. Table S1. List of all 60 test compounds and CAS numbers in HCA screening. Table S2. List of all 60 test compounds detail information. [file 13619_2022_148_MOESM1_ESM.zip › Xiaoshan HLO Supplemental Tables(20221018)R2.docx]

**Supplementary Table**

**Table S1** List of all 60 test compounds and CAS numbers in HCA screening.

**
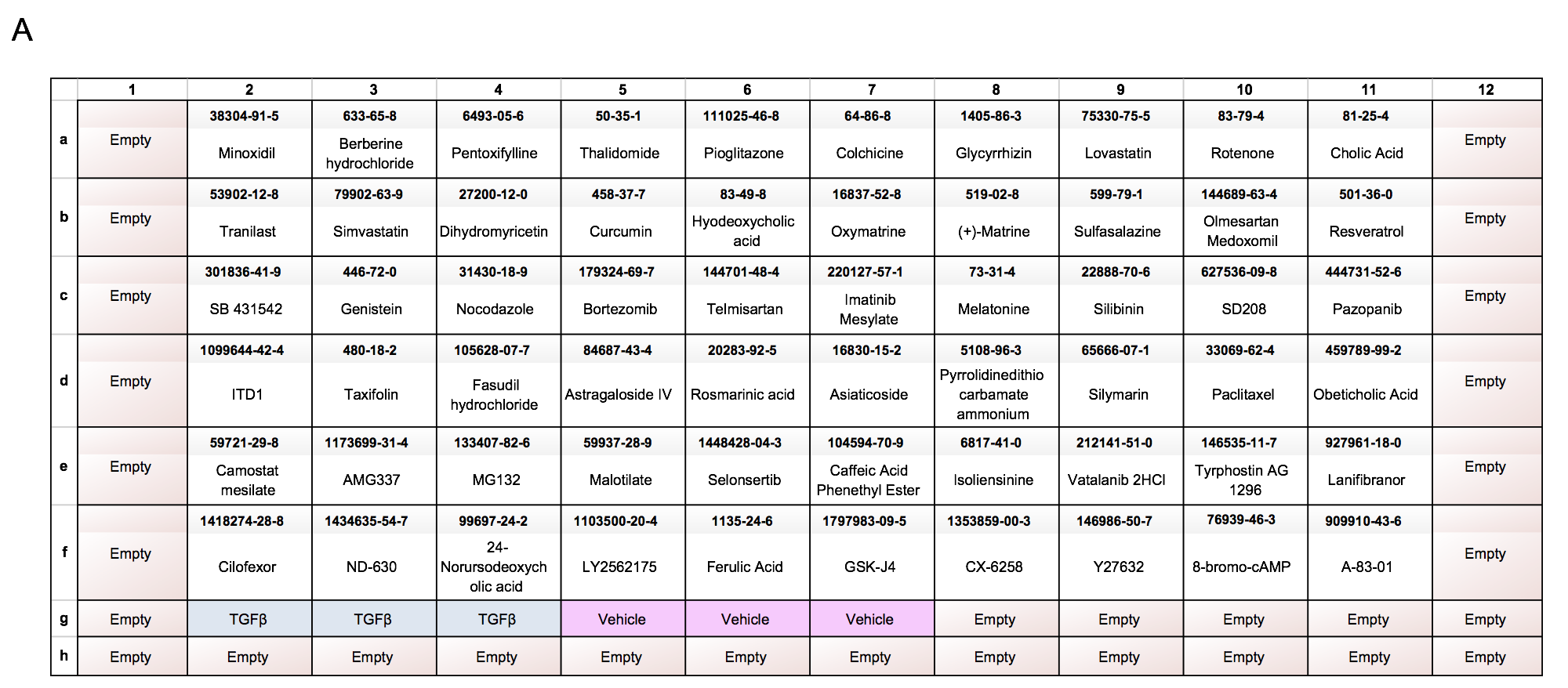
**

**Table S2** List of all 60 test compounds detail information.

| **Index** | **NAME** | **CAS** | **Pathways** | **Target** | **Bioactivity** | **Reference** | | | |
| --- | --- | --- | --- | --- | --- | --- | --- | --- | --- |
| 1 | Minoxidil | 38304-91-5 | Immunology/Inflammation; Membrane transporter/Ion channel; Neuroscience | COX inhibitor; Potassium channel antagonist | Minoxidil is an orally administered vasodilator with hair growth stimulatory and antihypertensive effects. | (Knitlova et al. 2021) |  | |  |
| 2 | Berberine hydrochloride | 633-65-8 | Microbiology& Virology | Antibiotic | Berberine hydrochloride is an alkaloid from Hydrastis canadensis L., Berberidaceae and also found in many other plants. It is relatively toxic to parenterally but has been used orally for various fungal and parasitic infections and an anti-diarrheal. | (Bai et al. 2020) |  | |  |
| 3 | Pentoxifylline | 6493-05-6 | GPCR/G Protein; Metabolism | Adenosine receptor antagonist; PDE inhibitor | Pentoxifylline modulates immunologic activity by stimulating cytokine production. Pentoxifylline is a methylxanthine derivative that inhibits phosphodiesterase and affects blood rheology. It also inhibits platelet aggregation and improves blood flow by increasing erythrocyte and leukocyte flexibility. | (Magnusson et al. 2009) |  | |  |
| 4 | Thalidomide | 50-35-1 | Apoptosis | TNF inhibitor | Thalidomide is a synthetic derivative of glutamic acid (alpha-phthalimido-glutarimide) with teratogenic, immunomodulatory, anti-inflammatory and anti-angiogenic properties. | (Yndestad et al. 2006) |  | |  |
| 5 | Pioglitazone | 111025-46-8 | Metabolism; DNA damage/DNA repair | PPAR agonist | Pioglitazone is an insulin sensitizing agent and thiazolidinedione that is indicated for the treatment of type 2 diabetes. Pioglitazone has been linked to rare instances of acute liver injury. | (Musso et al. 2017) |  | |  |
| 6 | Colchicine | 64-86-8 | Cytoskeletal signaling | Microtubule associated | Colchicine is a tubulin inhibitor and blocks polymerization of microtubules by binding to tubulin. | (Nikolaidis et al. 2006) |  |  |  |
| 7 | Glycyrrhizin | 1405-86-3 | Metabolism; Neuroscience | Dehydrogenase inhibitor; MAO | Glycyrrhizic acid is a triterpenoid saponinl with anti-tumor and anti-diabetic activities. | (Pun et al. 2021) |  |  |  |
| 8 | Lovastatin | 75330-75-5 | Metabolism | HMG-CoA reductase inhibitor | Lovastatin is an HMG-CoA reductase inhibitor, used for lowering cholesterol. | (Hamblin et al. 2014) |  |  |  |
| 9 | Rotenone | 83-79-4 | Apoptosis; Metabolism | Dehydrogenase inhibitor; p53 activator | Rotenone is a botanical insecticide that is an inhibitor of mitochondrial electron transport. Rotenone is widely distributed in the leguminosae. | (Sun et al. 2014) |  |  |  |
| 10 | Cholic acid | 81-25-4 | Metabolism | Endogenous metabolite | Cholic acid is a major primary bile acid produced in the liver and usually conjugated with glycine or taurine. It facilitates fat absorption and cholesterol excretion. | (Wu et al. 2015) |  |  |  |
| 11 | Tranilast | 53902-12-8 | GPCR/G protein | Angiotensin receptor | Tranilast, an antiallergic drug, suppresses lipid mediator and cytokine release from inflammatory cells, therefore utilized in the treatment of allergic disorders. | (Suzawa et al. 1992) |  | |  |
| 12 | Simvastatin | 79902-63-9 | Metabolism | HMG-CoA reductase inhibitor | Simvastatin is a hydroxymethylglutaryl-coA (HMG-CoA) reductase inhibitor. | (Davis et al. 2015) |  | |  |
| 13 | Dihydromyricetin | 27200-12-0 | Membrane transporter/Ion channel; Neuroscience; PI3K/Akt/mTOR signaling | GABA receptor agonist; mTOR | Dihydromyricetin is a natural antioxidant flavonoid from Ampelopsis grossedentata. Dihydromyricetin can activate autophagy by inhibiting mTOR signaling. | (Zhou et al. 2021) |  |  |  |
| 14 | Curcumin | 458-37-7 | Chromatin/Epigenetic; Others | Epigenetic reader domain inhibitor | Curcumin is a phytopolylphenol pigment isolated from the plant Curcuma longa, commonly known as turmeric, with a variety of pharmacologic properties. Curcumin blocks the formation of reactive oxygen species, possesses anti-inflammatory properties as a result of inhibition of cyclooxygenases (COX) and other enzymes involved in inflammation; and disrupts cell signal transduction by various mechanisms including inhibition of protein kinase C. These effects may play a role in the agent's observed antineoplastic properties, which include inhibition of tumor cell proliferation and suppression of chemically induced carcinogenesis and tumor growth in animal models of cancer. | (Gao et al. 2013) |  |  |  |
| 15 | Hyodeoxycholic acid | 83-49-8 | Metabolism; GPCR/G Protein | FXR antagonist; GPCR19 | Hyodeoxycholic Acid has been used in trials studying the treatment of Hypercholesterolemia. | (Shih et al. 2013) |  |  |  |
| 16 | Oxymatrine | 16837-52-8 | Autophagy | Autophagy inhibitor | Oxymatrine is an alkaloid isolated from Sophora flavescens, used as the antibiotic. It is a traditional Chinese medicine used in the treatment against hepatitis B virus. It can also inhibit iNOS expression and TGFβ/ Smad pathway. | (Xiang et al. 2002) |  |  |  |
| 17 | (+)-Matrine * | 519-02-8 | Endocrinology/Hormones; GPCR/G protein; Neuroscience | Opioid receptor agonist | Matrine, an alkaloid isolated from the Sophora genus, acts as a kappa opioid receptor agonist. | (Liu et al. 2017) |  |  |  |
| 18 | Sulfasalazine | 599-79-1 | Immunology/Inflammation; NF-κb; Neuroscience | COX inhibitor; NF-κB inhibitor | Sulfasalazine is a synthetic salicylic acid derivative with affinity for connective tissues containing elastin and formulated as a prodrug | (Gadangi et al. 1996) |  |  |  |
| 19 | Olmesartan medoxomil | 144689-63-4 | Endocrinology/Hormones | RAAS inhibitor | Olmesartan medoxomil is an angiotensin II type 1 receptor blocker that is used to manage hypertension. | (Kurikawa et al. 2003) |  |  |  |
| 20 | Resveratrol | 501-36-0 | Chromatin/Epigenetic; immunology/Inflammation; DNA damage/DNA repair; Metabolism; NF-κb; Neuroscience | COX; DNA/RNA Synthesis; IκB/IKK; Lipoxygenase; NADPH; Sirtuin | Resveratrol is a polyphenolic phytoalexin with antioxidant and chemopreventive activities. It has a wide spectrum of targets including COX, SIRT, LOC, etc. | (Kessoku et al. 2016) |  |  |  |
| 21 | SB 431542 | 301836-41-9 | Angiogenesis; Tyrosine Kinase/Adaptors; Stem cells | ALK | SB-431542 is a potent and selective inhibitor of ALK5 and is also an inhibitor of ALK4 and ALK7. | (Callahan et al. 2002) |  |  |  |
| 22 | Genistein | 446-72-0 | Angiogenesis; JAK/STAT signaling; Tyrosine kinase/Adaptors | EGFR antagonist | Genistein is an isoflavonoid derived from soy products. It inhibits protein-tyrosine kinase and topoisomerase-II, with antineoplastic and antitumor activities. | (Ning et al. 2020) |  |  |  |
| 23 | Nocodazole | 31430-18-9 | Angiogenesis; Cytoskeletal signaling; Tyrosine kinase/Adaptors | Bcr-Abl inhibitor; Microtubule Associated inhibitor | Nocodazole is a synthetic inhibitor of microtubule polymerization. Nocodazole binds to beta-tubulin and disrupts microtubule assembly/disassembly dynamics. | (Park et al. 2012) |  |  |  |
| 24 | Bortezomib | 179324-69-7 | Proteases/Proteasome; Ubiquitination | Proteasome | Bortezomib is a potent 20S proteasome inhibitor. | (Penke et al. 2022) |  |  |  |
| 25 | Telmisartan | 144701-48-4 | Endocrinology/Hormones | RAAS inhibitor | Telmisartan is an angiotensin 2 receptor blocker. The mechanism of action of telmisartan is as an Angiotensin 2 receptor antagonist. | (Zheng et al. 2022) |  |  |  |
| 26 | Imatinib mesylate* | 220127-57-1 | Angiogenesis; Tyrosine Kinase/Adaptors; Cytoskeletal signaling; chromatin/Epigenetic | Bcr-Abl; c-Kit; PDGFR | Imatinib mesylate is a tyrosine kinase receptor inhibitor with antineoplastic activity. | (Guan et al. 2021) |  |  |  |
| 27 | Melatonine | 73-31-4 | Endocrinology/Hormones; GPCR/G protein; Metabolism; Neuroscience | CaMK agonist; Estrogen/progestogen receptor antagonist; Melatonin receptor agonist; MPO inhibitor; ROR agonist | Melatonin is a biogenic amine that is found in animals and plants. In mammals, melatonin is produced by the pineal gland. Its secretion increases in darkness and decreases during exposure to light. Melatonin is implicated in the regulation of sleep, mood, and reproduction. Melatonin is also an effective antioxidant. | (Reiter et al. 2000) |  |  |  |
| 28 | Silibinin | 22888-70-6 | Autophagy | Autophagy | Silibinin itself is a mixture of two diastereomers, silybin A and silybin B, in approximately equimolar ratio. Both *in vitro* and animal research suggest that silibinin has hepatoprotective (antihepatotoxic) properties that protect liver cells against toxins. | (Al-Anati et al. 2009) |  |  |  |
| 29 | SD208* | 627536-09-8 | Stem cells | TGFβ/Smad inhibitor | SD208, a selective TGFβRI (ALK5) inhibitor, is >100-fold selectivity over TGFβRII. | (Uhl et al. 2004) |  |  |  |
| 30 | Pazopanib | 444731-52-6 | Angiogenesis; Tyrosine kinase/Adaptors; chromatin/Epigenetic | c-Kit inhibitor; PDGFR inhibitor; VEGFR inhibitor | Pazopanib, a small molecule inhibitor, inhibits multiple protein tyrosine kinases with potential antineoplastic activity. Pazopanib selectively inhibits VEGFR-1, -2 and -3, c-kit and PDGF-R, which may result in inhibition of angiogenesis in tumors in which these receptors are upregulated. | (Elshal et al. 2015) |  |  |  |
| 31 | ITD1 | 1099644-42-4 | Stem cells | TGFβ/Smad inhibitor | ITD-1 is a potent and highly selective TGFβ pathway inhibitor. | (Willems et al. 2012) |  |  |  |
| 32 | Taxifolin | 480-18-2 | GPCR/G Protein; Angiogenesis; Apoptosis; Proteases/Proteasome; Tyrosine kinase/Adaptors | Adrenergic Receptor antagonist; TNF inhibitor; Tyrosinase inhibitor; VEGFR inhibitor | Taxifolin is a flavonoid in many plants such as Taxus chinensis, Siberian larch, Cedrus deodara and so on. | (Ren et al. 2020) |  |  |  |
| 33 | Fasudil hydrochloride | 105628-07-7 | Cell Cycle/Checkpoint; Apoptosis; Cytoskeletal Signaling; GPCR/G Protein; Stem Cells | PKA inhibitor; PKC inhibitor; ROCK inhibitor; Serine/threonin kinase inhibitor | Fasudil Hydrochloride is a potent inhibitor of ROCK1, PKA, PKC, and MLCK. | (Zhou et al. 2014) |  |  |  |
| 34 | Astragaloside IV | 84687-43-4 | Endocrinology/Hormones | Estrogen/progestogen Receptor antagonist | Astragaloside IV, an active component isolated from Astragalus membranaceus, suppresses the activation of ERK1/2 and JNK, and downregulates matrix metalloproteases (MMP)-2, (MMP)-9 in MDA-MB-231 breast cancer cells. It dose-dependently inhibits human adenovirus type 3 (HAdV-3) in A549 cells. It inhibits replication of HAdV-3 and decreases HAdV-3-induced apoptosis. It has diverse protective effects for the cardiovascular, immune, digestive, and nervous systems. | (Li et al. 2017) |  |  |  |
| 35 | Rosmarinic acid | 20283-92-5 | Metabolism; Neuroscience; NF-κb | MAO inhibitor; Transferase inhibitor; IκB/IKK | Rosmarinic acid has shown to contain antioxidant, anti-inflammatory and antimicrobial activities. Possesses promising physiological actions related to cognitive performance, Alzheimer′s disease prevention, kideney disease treatment, cardioprotection and cancer chemoprevention. | (El-Lakkany et al. 2017) |  |  |  |
| 36 | Asiaticoside* | 16830-15-2 | MAPK; Stem cells | p38 MAPK inhibitor; TGFβ /Smad inhibitor | Asiaticoside (Madecassol) is the active chemical component of the plant Centella asiatica. Asiaticoside is used to study potential treatments for wounds and burns. | (Luo et al. 2015) |  |  |  |
| 37 | Pyrrolidinedithiocarbamate ammonium* | 5108-96-3 | NF-κb | NF-κB inhibitor | Pyrrolidinedithiocarbamate ammonium, a selective NF-κB inhibitor, inhibits translation of nitric oxide synthase mRNA to prevent induction. | (Qin et al. 2014) |  |  |  |
| 38 | Silymarin* | 65666-07-1 | Others | Others | Silymarin (Silybin B) is a polyphenolic flavonoid that extracts from the milk thistle or seeds of Silybum marianum. It is used in the prevention and treatment of liver diseases. | (Clichici et al. 2015) |  |  |  |
| 39 | Paclitaxel | 33069-62-4 | Cytoskeletal signaling | Microtubule | Paclitaxel is a cyclodecane isolated from the bark of the Pacific yew tree. It stabilizes microtubules in their polymerized form leading to cell death. | (Sharawy et al. 2018) |  | |  |
| 40 | Obeticholic acid | 459789-99-2 | Metabolism | FXR agonist | Obeticholic acid is a farnesoid X receptor (FXR) agonist. | (Zhou et al. 2019) |  |  |  |
| 41 | Camostat mesilate* | 59721-29-8 | Membrane transporter/Ion channel | Sodium channel inhibitor | Camostat is a trypsin-like protease inhibitor and inhibits airway epithelial sodium channel (ENaC) function. | (Okuno et al. 2001) |  |  |  |
| 42 | AMG337 | 1173699-31-4 | Tyrosine kinase/Adaptors | c-Met/HGFR inhibitor | AMG-337 is an effective and highly specific ATP-competitive MET kinase inhibitor. In enzymatic assays, AMG-337 inhibits MET kinase activity. | (Hughes et al. 2016) |  |  |  |
| 43 | MG132 | 133407-82-6 | Proteases/Proteasome; Ubiquitination | Proteasome, Cysteine protease | MG-132 is a potent cell-permeable 20S proteasome inhibitor. It also inhibits calpain. | (Han et al. 2019) |  |  |  |
| 44 | Malotilate | 59937-28-9 | Metabolism | Lipoxygenase | Malotilate is a medicine used for the therapy of liver cirrhosis. | (Ryhanen et al. 1996) |  |  |  |
| 45 | Selonsertib | 1448428-04-3 | Apoptosis | ASK inhibitor | Selonsertib is an orally bioavailable inhibitor of apoptosis signal-regulating kinase 1 (ASK1), with potential anti-inflammatory, antineoplastic and anti-fibrotic activities. | (Yoon et al. 2020) |  |  |  |
| 46 | Caffeic Acid Phenethyl Ester | 104594-70-9 | NF-κb | NF-κB inhibitor | Caffeic acid phenethyl ester (CAPE) inhibits the activation of nuclear transcription factor NF-κB and may suppress p70S6K and Akt-driven signaling pathways, with antineoplastic, cytoprotective and immunomodulating activities. In addition, CAPE inhibits PDGF-induced proliferation of vascular smooth muscle cells through the activation of p38 mitogen-activated protein kinase (MAPK) and hypoxia inducible factor-1α and subsequent induction of heme oxygenase-1 (HO-1). | (Natarajan et al. 1996) |  |  |  |
| 47 | Isoliensinine | 6817-41-0 | oxidation-reduction | Antioxidant | Isoliensinine is a natural phenolic bisbenzyltetrahydroisoquinoline alkaloid, has received considerable attention for its potential biological effects such as antioxidant and anti-HIV activities. Isoliensinine possesses an anti-proliferative effect, which is related to the decrease of the overexpression of growth factors PDGF-beta, bFGF, proto-oncogene c-fos, c-myc and hsp7. | (Zhang et al. 2015) |  |  |  |
| 48 | Vatalanib 2HCl | 212141-51-0 | Angiogenesis; Tyrosine kinase/Adaptors; Chromatin/Epigenetic | PDGFR inhibitor; VEGFR inhibitor | Vatalanib is an inhibitor of VEGFR2/KDR. It exhibits less effective against VEGFR1/Flt-1 and 18-fold against VEGFR3/Flt-4. | (Kong et al. 2017) |  |  |  |
| 49 | Tyrphostin AG 1296* | 146535-11-7 | Angiogenesis; Tyrosine kinase/Adaptors; Chromatin/Epigenetic | PDGFR inhibitor; c-Kit; FGFR | Tyrphostin AG 1296 is an inhibitor of PDGFR, no activity to EGFR. | (Kovalenko et al. 1994) |  |  |  |
| 50 | Lanifibranor | 927961-18-0 | Metabolism; DNA Damage/DNA Repair | PPAR | Lanifibranor is an agonist of peroxisome proliferator-activated receptors (PPARs) with EC50 values of 1,537, 866, and 206 nM for human recombinant PPARα, PPARβ, and PPARγ, respectively, for transactivation activity. | (Ruzehaji et al. 2016) | |  |  |
| 51 | Cilofexor* | 1418274-28-8 | Metabolism | FXR | Cilofexor inhibits binding of a synthetic peptide. Cilofexor is a farnesoid X receptor (FXR) agonist. | (Patel et al. 2020) |  |  |  |
| 52 | ND-630 | 1434635-54-7 | Proteases/Proteasome | Acetyl-CoA carboxylase | ND-630 is an inhibitor of acetyl-CoA carboxylase (ACC) dimerization that inhibits human ACC1 and ACC2 activity (IC50s of 2.1 and 6.1 nM, respectively) | (Harriman et al. 2016) |  |  |  |
| 53 | 24-Norursodeoxycholic acid | 99697-24-2 | Others | Others | 24-norursodeoxycholic acid is a side chain-shortened C23 homolog of UDCA. It has shown potent anti-inflammatory, anti-cholestatic, and anti-fibrotic properties. It is a usodeoxycholic acid derivative. | (Buko et al. 2014) |  |  |  |
| 54 | LY2562175 | 1103500-20-4 | Autophagy; Metabolism | Autophagy; FXR | LY2562175 is an effective and selective FXR agonist. | (Miyata et al. 2021) |  |  |  |
| 55 | Ferulic Acid | 1135-24-6 | Autophagy; Metabolism | FGFR1 | Ferulic Acid is a highly abundant phenolic phytochemical and a type of organic compound found in the Ferula assafoetida L. or Ligusticum chuanxiong. It including antioxidant, hepatoprotective, anticarcinogenic, anti-inflammatory and preventing Alzheimer’s disease and cardiovascular diseases. | (Wu et al. 2021) |  |  |  |
| 56 | GSK-J4 | 1797983-09-5 | Chromosome and associated proteins | H3K27 histone demethylase JMJD3 and UTX | GSK-J4 is a cell permeable prodrug of GSK J1, which is the first selective inhibitor of the H3K27 histone demethylase JMJD3 and UTX in a cell-free assay and inactive against a panel of demethylases of the JMJ family. | (Chen et al. 2019) |  |  |  |
| 57 | CX-6258 | 1353859-00-3 | JAK-STAT signaling pathway; AGE-RAGE signaling pathway in diabetic complications | Pim 1/2/3 kinase | CX-6258 is an orally valid Pim 1/2/3 kinase inhibitor. It has good biological activity and kinase specificity. | (Haddach et al. 2012) |  |  |  |
| 58 | Y27632 | 146986-50-7 | cGMP-PKG signaling pathway; cAMP signaling pathway | ROCK1/ROCK2 | Y-27632 is an inhibitor of Rho-associated protein kinase. It inhibits calcium sensitization to affect smooth muscle relaxation. | (Wei et al. 2020) |  |  |  |
| 59 | 8-bromo-cAMP | 76939-46-3 | cAMP-PKA signaling pathway | PKA | 8-Bromo-cAMP is a long-acting derivative of cyclic AMP. It is an activator of cyclic AMP-dependent protein kinase, but resistant to degradation by cyclic AMP phosphodiesterase. | (Insel et al. 2012) |  |  |  |
| 60 | A-83-01 | 909910-43-6 | TGF-β signaling pathway | ALK5 kinase | A83-01 is a potent inhibitor of ALK5 kinase, type I activin/nodal receptor ALK4 and type I nodal receptor ALK7. | (Tojo et al. 2005) |  | |  |

*represent the compound that was grouped together with three vehicle groups by tSNE.

**References**

Al-Anati L, Essid E, Reinehr R, Petzinger E. Silibinin protects OTA-mediated TNF-alpha release from perfused rat livers and isolated rat Kupffer cells. Mol Nutr Food Res. 2009;53:460-6. <https://10.1002/mnfr.200800110>.

Bai F, Tao H, Wang P, Wang L, Zhou X, Wang F, et al. Berberine hydrochloride inhibits inflammation and fibrosis after canalicular laceration repair in rabbits. Life Sci. 2020;261:118479. <https://10.1016/j.lfs.2020.118479>.

Buko VU, Lukivskaya OY, Naruta EE, Belonovskaya EB, Tauschel HD. Protective effects of norursodeoxycholic acid versus ursodeoxycholic acid on thioacetamide-induced rat liver fibrosis. J Clin Exp Hepatol. 2014;4:293-301. <https://10.1016/j.jceh.2014.02.001>.

Callahan JF, Burgess JL, Fornwald JA, Gaster LM, Harling JD, Harrington FP, et al. Identification of novel inhibitors of the transforming growth factor beta1 (TGF-beta1) type 1 receptor (ALK5). J Med Chem. 2002;45:999-1001. <https://10.1021/jm010493y>.

Chen H, Huang YX, Zhu XQ, Liu C, Yuan YM, Su H, et al. Histone demethylase UTX is a therapeutic target for diabetic kidney disease. J Physiol-London. 2019;597:1643-60. <https://10.1113/Jp277367>.

Clichici S, Olteanu D, Nagy AL, Oros A, Filip A, Mircea PA. Silymarin inhibits the progression of fibrosis in the early stages of liver injury in CCl(4)-treated rats. J Med Food. 2015;18:290-8. <https://10.1089/jmf.2013.0179>.

Davis ME, Korn MA, Gumucio JP, Harning JA, Saripalli AL, Bedi A, et al. Simvastatin reduces fibrosis and protects against muscle weakness after massive rotator cuff tear. J Shoulder Elbow Surg. 2015;24:280-7. <https://10.1016/j.jse.2014.06.048>.

El-Lakkany NM, El-Maadawy WH, Seif El-Din SH, Hammam OA, Mohamed SH, Ezzat SM, et al. Rosmarinic acid attenuates hepatic fibrogenesis via suppression of hepatic stellate cell activation/proliferation and induction of apoptosis. Asian Pac J Trop Med. 2017;10:444-53. <https://10.1016/j.apjtm.2017.05.012>.

Elshal M, Abu-Elsaad N, El-Karef A, Ibrahim TM. The multi-kinase inhibitor pazopanib targets hepatic stellate cell activation and apoptosis alleviating progression of liver fibrosis. Naunyn Schmiedebergs Arch Pharmacol. 2015;388:1293-304. <https://10.1007/s00210-015-1157-7>.

Gadangi P, Longaker M, Naime D, Levin RI, Recht PA, Montesinos MC, et al. The anti-inflammatory mechanism of sulfasalazine is related to adenosine release at inflamed sites. J Immunol. 1996;156:1937-41.

Gao S, Duan X, Wang X, Dong D, Liu D, Li X, et al. Curcumin attenuates arsenic-induced hepatic injuries and oxidative stress in experimental mice through activation of Nrf2 pathway, promotion of arsenic methylation and urinary excretion. Food Chem Toxicol. 2013;59:739-47. <https://10.1016/j.fct.2013.07.032>.

Guan Y, Enejder A, Wang M, Fang Z, Cui L, Chen SY, et al. A human multi-lineage hepatic organoid model for liver fibrosis. Nat Commun. 2021;12:6138. <https://10.1038/s41467-021-26410-9>.

Haddach M, Michaux J, Schwaebe MK, Pierre F, O'Brien SE, Borsan C, et al. Discovery of CX-6258. A potent, selective, and orally efficacious pan-Pim kinases inhibitor. Acs Med Chem Lett. 2012;3:135-9. <https://10.1021/ml200259q>.

Hamblin MJ, Eberlein M, Black K, Hallowell R, Collins S, Chan-Li Y, et al. Lovastatin inhibits low molecular weight hyaluronan induced chemokine expression via LFA-1 and decreases bleomycin-induced pulmonary fibrosis. Int J Biomed Sci. 2014;10:146-57.

Han L, Zhu B, Chen H, Jin Y, Liu J, Wang W. Proteasome inhibitor MG132 inhibits the process of renal interstitial fibrosis. Exp Ther Med. 2019;17:2953-62. <https://10.3892/etm.2019.7329>.

Harriman G, Greenwood J, Bhat S, Huang X, Wang R, Paul D, et al. Acetyl-CoA carboxylase inhibition by ND-630 reduces hepatic steatosis, improves insulin sensitivity, and modulates dyslipidemia in rats. Proc Natl Acad Sci U S A. 2016;113:E1796-805. <https://10.1073/pnas.1520686113>.

Hughes PE, Rex K, Caenepeel S, Yang Y, Zhang Y, Broome MA, et al. *In vitro* and *in vivo* activity of AMG 337, a potent and selective MET kinase inhibitor, in MET-dependent cancer models. Mol Cancer Ther. 2016;15:1568-79. <https://10.1158/1535-7163.MCT-15-0871>.

Insel PA, Murray F, Yokoyama U, Romano S, Yun H, Brown L, et al. cAMP and Epac in the regulation of tissue fibrosis. Br J Pharmacol. 2012;166:447-56. <https://10.1111/j.1476-5381.2012.01847.x>.

Kessoku T, Imajo K, Honda Y, Kato T, Ogawa Y, Tomeno W, et al. Resveratrol ameliorates fibrosis and inflammation in a mouse model of nonalcoholic steatohepatitis. Sci Rep. 2016;6:22251. <https://10.1038/srep22251>.

Knitlova J, Doubkova M, Plencner M, Vondrasek D, Eckhardt A, Ostadal M, et al. Minoxidil decreases collagen I deposition and tissue-like contraction in clubfoot-derived cells: a way to improve conservative treatment of relapsed clubfoot? Connect Tissue Res. 2021;62:554-69. <https://10.1080/03008207.2020.1816992>.

Kong LJ, Li H, Du YJ, Pei FH, Hu Y, Zhao LL, et al. Vatalanib, a tyrosine kinase inhibitor, decreases hepatic fibrosis and sinusoidal capillarization in CCl4-induced fibrotic mice. Mol Med Rep. 2017;15:2604-10. <https://10.3892/mmr.2017.6325>.

Kovalenko M, Gazit A, Bohmer A, Rorsman C, Ronnstrand L, Heldin CH, et al. Selective platelet-derived growth-factor receptor kinase blockers reverse sis-transformation. Cancer Research. 1994;54:6106-14.

Kurikawa N, Suga M, Kuroda S, Yamada K, Ishikawa H. An angiotensin II type 1 receptor antagonist, olmesartan medoxomil, improves experimental liver fibrosis by suppression of proliferation and collagen synthesis in activated hepatic stellate cells. Br J Pharmacol. 2003;139:1085-94. <https://10.1038/sj.bjp.0705339>.

Li M, Li H, Fang F, Deng X, Ma S. Astragaloside IV attenuates cognitive impairments induced by transient cerebral ischemia and reperfusion in mice via anti-inflammatory mechanisms. Neurosci Lett. 2017;639:114-9. <https://10.1016/j.neulet.2016.12.046>.

Liu Z, Zhang Y, Tang Z, Xu J, Ma M, Pan S, et al. Matrine attenuates cardiac fibrosis by affecting ATF6 signaling pathway in diabetic cardiomyopathy. Eur J Pharmacol. 2017;804:21-30. <https://10.1016/j.ejphar.2017.03.061>.

Luo Y, Fu CF, Wang ZY, Zhang Z, Wang HX, Liu Y. Asiaticoside attenuates the effects of spinal cord injury through antioxidant and anti-inflammatory effects, and inhibition of the p38-MAPK mechanism. Mol Med Rep. 2015;12:8294-300. <https://10.3892/mmr.2015.4425>.

Magnusson M, Hoglund P, Johansson K, Jonsson C, Killander F, Malmstrom P, et al. Pentoxifylline and vitamin E treatment for prevention of radiation-induced side-effects in women with breast cancer: a phase two, double-blind, placebo-controlled randomised clinical trial (Ptx-5). Eur J Cancer. 2009;45:2488-95. <https://10.1016/j.ejca.2009.05.015>.

Miyata S, Kawashima Y, Sakai M, Matsubayashi M, Motoki K, Miyajima Y, et al. Discovery, optimization, and evaluation of non-bile acid FXR/TGR5 dual agonists. Sci Rep. 2021;11:9196. <https://10.1038/s41598-021-88493-0>.

Musso G, Cassader M, Paschetta E, Gambino R. Pioglitazone for advanced fibrosis in nonalcoholic steatohepatitis: New evidence, new challenges. Hepatology. 2017;65:1058-61. <https://10.1002/hep.28960>.

Natarajan K, Singh S, Burke TR, Grunberger D, Aggarwal BB. Caffeic acid phenethyl ester is a potent and specific inhibitor of activation of nuclear transcription factor NF-kappa B. P Natl Acad Sci USA. 1996;93:9090-5. [https://doi/](https://DOI) 10.1073/pnas.93.17.9090.

Nikolaidis N, Kountouras J, Giouleme O, Tzarou V, Chatzizisi O, Patsiaoura K, et al. Colchicine treatment of liver fibrosis. Hepatogastroenterology. 2006;53:281-5.

Ning Y, Chen J, Shi Y, Song N, Yu X, Fang Y, et al. Genistein ameliorates renal fibrosis through regulation snail via m6A RNA demethylase ALKBH5. Front Pharmacol. 2020;11:579265. <https://10.3389/fphar.2020.579265>.

Okuno M, Akita K, Moriwaki H, Kawada N, Ikeda K, Kaneda K, et al. Prevention of rat hepatic fibrosis by the protease inhibitor, camostat mesilate, via reduced generation of active TGF-beta. Gastroenterology. 2001;120:1784-800. <https://10.1053/gast.2001.24832>.

Park H, Hong S, Hong S. Nocodazole is a high-affinity ligand for the cancer-related kinases ABL, c-KIT, BRAF, and MEK. Chemmedchem. 2012;7:53-6. <https://10.1002/cmdc.201100410>.

Patel K, Harrison SA, Elkhashab M, Trotter JF, Herring R, Rojter SE, et al. Cilofexor, a nonsteroidal FXR agonist, in patients with noncirrhotic NASH: A phase 2 randomized controlled trial. Hepatology. 2020;72:58-71. <https://10.1002/hep.31205>.

Penke LRK, Speth J, Wettlaufer S, Draijer C, Peters-Golden M. Bortezomib inhibits lung fibrosis and fibroblast activation without proteasome inhibition. Am J Respir Cell Mol Biol. 2022;66:23-37. <https://10.1165/rcmb.2021-0112OC>.

Pun CK, Huang HC, Chang CC, Chuang CL, Yen CH, Hsu SJ, et al. Glycyrrhizin attenuates portal hypertension and collateral shunting via inhibition of extrahepatic angiogenesis in cirrhotic rats. Int J Mol Sci. 2021;22. <https://10.3390/ijms22147662>.

Qin JD, Cao ZH, Li XF, Kang XL, Xue Y, Li YL, et al. Effect of ammonium pyrrolidine dithiocarbamate (PDTC) on NF-kappaB activation and CYP2E1 content of rats with immunological liver injury. Pharm Biol. 2014;52:1460-6. <https://10.3109/13880209.2014.898075>.

Reiter RJ, Tan DX, Osuna C, Gitto E. Actions of melatonin in the reduction of oxidative stress - A review. J Biomed Sci. 2000;7:444-58. [https://doi/](https://Doi) 10.1007/Bf02253360.

Ren L, Guo HN, Yang J, Guo XY, Wei YS, Yang Z. Dissecting efficacy and metabolic characteristic mechanism of taxifolin on renal fibrosis by multivariate approach and ultra-performance liquid chromatography coupled with mass spectrometry-based metabolomics strategy. Front Pharmacol. 2020;11:608511. <https://10.3389/fphar.2020.608511>.

Ruzehaji N, Frantz C, Ponsoye M, Avouac J, Pezet S, Guilbert T, et al. Pan PPAR agonist IVA337 is effective in prevention and treatment of experimental skin fibrosis. Ann Rheum Dis. 2016;75:2175-83. <https://10.1136/annrheumdis-2015-208029>.

Ryhanen L, Stenback F, Ala-Kokko L, Savolainen ER. The effect of malotilate on type III and type IV collagen, laminin and fibronectin metabolism in dimethylnitrosamine-induced liver fibrosis in the rat. J Hepatol. 1996;24:238-45. <https://10.1016/s0168-8278(96)80035-3>.

Sharawy MH, Abdel-Rahman N, Megahed N, El-Awady MS. Paclitaxel alleviates liver fibrosis induced by bile duct ligation in rats: Role of TGF-beta1, IL-10 and c-Myc. Life Sci. 2018;211:245-51. <https://10.1016/j.lfs.2018.09.037>.

Shih DM, Shaposhnik Z, Meng Y, Rosales M, Wang X, Wu J, et al. Hyodeoxycholic acid improves HDL function and inhibits atherosclerotic lesion formation in LDLR-knockout mice. FASEB J. 2013;27:3805-17. <https://10.1096/fj.12-223008>.

Sun Y, Zhang Y, Zhao D, Ding G, Huang S, Zhang A, et al. Rotenone remarkably attenuates oxidative stress, inflammation, and fibrosis in chronic obstructive uropathy. Mediators Inflamm. 2014;2014:670106. <https://10.1155/2014/670106>.

Suzawa H, Kikuchi S, Arai N, Koda A. The mechanism involved in the inhibitory action of tranilast on collagen biosynthesis of keloid fibroblasts. Jpn J Pharmacol. 1992;60:91-6. <https://10.1254/jjp.60.91>.

Tojo M, Hamashima Y, Hanyu A, Kajimoto T, Saitoh M, Miyazono K, et al. The ALK-5 inhibitor A-83-01 inhibits Smad signaling and epithelial-to-mesenchymal transition by transforming growth factor-beta. Cancer Science. 2005;96:791-800. <https://10.1111/j.1349-7006.2005.00103.x>.

Uhl M, Aulwurm S, Wischhusen J, Weiler M, Ma JY, Almirez R, et al. SD-208, a novel transforming growth factor beta receptor I kinase inhibitor, inhibits growth and invasiveness and enhances immunogenicity of murine and human glioma cells *in vitro* and *in vivo*. Cancer Res. 2004;64:7954-61. <https://10.1158/0008-5472.CAN-04-1013>.

Wei YH, Liao SL, Wang SH, Wang CC, Yang CH. Simvastatin and ROCK inhibitor Y-27632 inhibit myofibroblast differentiation of graves' ophthalmopathy-derived orbital fibroblasts via RhoA-mediated ERK and p38 signaling pathways. Front Endocrinol (Lausanne). 2020;11:607968. <https://10.3389/fendo.2020.607968>.

Willems E, Cabral-Teixeira J, Schade D, Cai W, Reeves P, Bushway PJ, et al. Small molecule-mediated TGF-beta type II receptor degradation promotes cardiomyogenesis in embryonic stem cells. Cell Stem Cell. 2012;11:242-52. <https://10.1016/j.stem.2012.04.025>.

Wu J, Xue X, Fan G, Gu Y, Zhou F, Zheng Q, et al. Ferulic acid ameliorates hepatic inflammation and fibrotic liver Injury by Inhibiting PTP1B activity and subsequent promoting AMPK phosphorylation. Front Pharmacol. 2021;12:754976. <https://10.3389/fphar.2021.754976>.

Wu Y, Wang Z, Liu G, Zeng X, Wang X, Gao Y, et al. Novel simvastatin-loaded nanoparticles based on cholic acid-core star-shaped PLGA for breast cancer treatment. J Biomed Nanotechnol. 2015;11:1247-60. <https://10.1166/jbn.2015.2068>.

Xiang X, Wang G, Cai X, Li Y. Effect of oxymatrine on murine fulminant hepatitis and hepatocyte apoptosis. Chin Med J (Engl). 2002;115:593-6.

Yndestad A, Vinge LE, Bjornerheim R, Ueland T, Wang JE, Froland SS, et al. Thalidomide attenuates the development of fibrosis during post-infarction myocardial remodelling in rats. Eur J Heart Fail. 2006;8:790-6. <https://10.1016/j.ejheart.2006.02.007>.

Yoon YC, Fang Z, Lee JE, Park JH, Ryu JK, Jung KH, et al. Selonsertib inhibits liver fibrosis via downregulation of ASK1/ MAPK pathway of hepatic stellate cells. Biomol Ther (Seoul). 2020;28:527-36. <https://10.4062/biomolther.2020.016>.

Zhang XY, Wang XY, Wu TT, Li BX, Liu TQ, Wang R, et al. Isoliensinine induces apoptosis in triple-negative human breast cancer cells through ROS generation and p38 MAPK/JNK activation. Sci Rep-Uk. 2015;5. [https://artn/](https://ARTN) 12579

10.1038/srep12579.

Zheng L, Zhao Z, Lin J, Li H, Wu G, Qi X, et al. Telmisartan relieves liver fibrosis and portal hypertension by improving vascular remodeling and sinusoidal dysfunction. Eur J Pharmacol. 2022;915:174713. <https://10.1016/j.ejphar.2021.174713>.

Zhou H, Fang C, Zhang L, Deng Y, Wang M, Meng F. Fasudil hydrochloride hydrate, a Rho-kinase inhibitor, ameliorates hepatic fibrosis in rats with type 2 diabetes. Chin Med J (Engl). 2014;127:225-31.

Zhou J, Huang N, Guo Y, Cui S, Ge C, He Q, et al. Combined obeticholic acid and apoptosis inhibitor treatment alleviates liver fibrosis. Acta Pharm Sin B. 2019;9:526-36. <https://10.1016/j.apsb.2018.11.004>.

Zhou X, Yu L, Zhou M, Hou P, Yi L, Mi M. Dihydromyricetin ameliorates liver fibrosis via inhibition of hepatic stellate cells by inducing autophagy and natural killer cell-mediated killing effect. Nutr Metab (Lond). 2021;18:64. <https://10.1186/s12986-021-00589-6>.
